# Multimodal Imaging of Hypoxia, Metabolism and pH in Feline Sarcoma Using Hyperpolarized ^13^C MRI and PET

**DOI:** 10.1002/advs.77830

**Published:** 2026-09-27

**Authors:** Martin Grashei, Tanja Groll, Stephanie Kahl, Silke Baer, Elisabeth Bliemsrieder, Frits H. A. van Heijster, Andre Wendlinger, Pascal Wodtke, Jorge Cabello, Susan Notohamiprodjo, Christian Lohrmann, Markus Schwaiger, Wolfgang A. Weber, Katja Steiger, Andrea Meyer‐Lindenberg, Johannes Hirschberger, Christine Baumgartner, Franz Schilling

**Affiliations:** ^1^ TUM School of Medicine and Health Department of Nuclear Medicine TUM University Hospital Technical University of Munich Munich Germany; ^2^ Comparative Experimental Pathology (CEP) Institute of Pathology TUM School of Medicine and Health Technical University of Munich Munich Germany; ^3^ Clinic of Small Animal Medicine LMU Munich Munich Germany; ^4^ TUM School of Medicine and Health Center for Preclinical Research TUM University Hospital Technical University of Munich Munich Germany; ^5^ German Cancer Consortium (DKTK) Partner Site Munich and German Cancer Research Center (DKFZ) Heidelberg Germany; ^6^ Clinic of Small Animal Surgery and Reproduction Centre of Clinical Veterinary Medicine LMU Munich Munich Germany; ^7^ Munich Institute of Biomedical Engineering Technical University of Munich Garching Germany

**Keywords:** cat, hyperpolarization, hypoxia, metabolism, PET/MRI, pH, sarcoma

## Abstract

Direct in vivo characterization of the interplay between tumor hypoxia, acidosis, and metabolic reprogramming remains a major translational challenge. Here, we establish a clinically deployable simultaneous PET/MRI workflow on a clinical 3 T system that combines hyperpolarized ^13^C MRI of [1‐^13^C]pyruvate to map metabolic flux, [1,5‐^13^C_2_,3,6,6,6‐D_4_]zymonic acid to probe extracellular pH, and [^18^F]FMISO PET to quantify hypoxia. Recruitment of domestic cats bearing sarcoma as a clinically relevant and comparative disease model yielded a highly heterogeneous study cohort. All modalities could be applied successfully and allow for simultaneous, coregistered mapping of acidification in spontaneous tumors in domestic cats for the first time, complemented by imaging of metabolism and hypoxia. Correlation of imaging parameters provides a multi‐dimensional assessment of inter‐ and intratumoral heterogeneity, which indicates distinct parameter correlation fingerprints for different subtypes. This multiparametric protocol allows for new insights into the metabolic program of sarcoma, suggesting tumor acidification to be coupled with hypoxia and increased lactate production. Moreover, pH imaging shows strong potential for assessing sarcoma aggressiveness and related subject outcomes. Together, these results establish a translationally deployable, simultaneous PET/MRI workflow for integrated imaging of tumor metabolism and microenvironment, which supports efforts to translate pH imaging for patient stratification and therapy monitoring.

## Graphical abstract

This work presents a clinically deployable simultaneous PET/MRI workflow which integrates hyperpolarized ^13^C imaging of metabolism and extracellular pH with [^18^F]FMISO PET to characterize hypoxia, acidosis, and metabolic reprogramming in feline sarcoma. This multiparametric approach reveals subtype‐specific heterogeneity, links acidification with hypoxia and lactate production, and highlights pH imaging as a promising biomarker for aggressiveness and patient stratification.

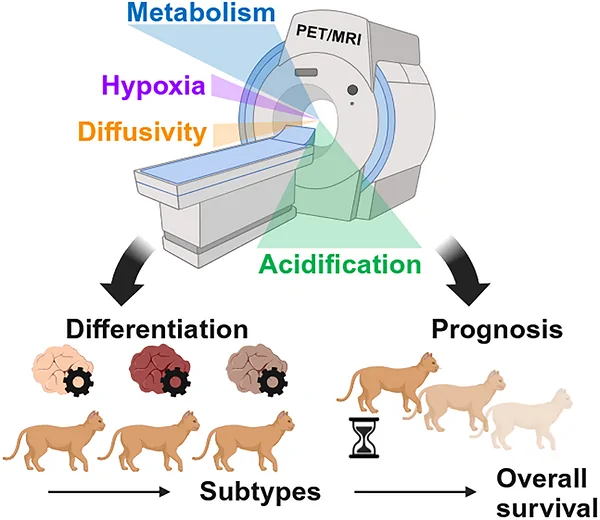

## 1 Introduction

Cancer cells often increase lactate production, as deregulation of metabolism is a hallmark of cancers [1]. Otto Warburg recognized early on that enhanced glycolysis and lactate formation can occur even in the presence of oxygen [2], yet hypoxia signaling acts manifold on metabolic pathways [3]. Lactate export can contribute to acidification of the extracellular space [4], which can be further exacerbated by multiple other mechanisms. Tumor acidity has been linked to proliferation, metastasis, and immune escape [5, 6, 7, 8]. Clinically, acidity is relevant because it shapes antitumor immunity [7] and may represent a modifiable vulnerability through microenvironment‐targeted conditioning strategies [9]. Together, hypoxia, lactate‐associated metabolism, and extracellular pH constitute key, interrelated parameters in tumor biology [10]. They are frequently treated as tightly coupled hallmarks of solid tumors, and several imaging studies have assessed pairs [11, 12, 13, 14, 15, 16, 17, 18] of these factors. However, a clinically transferable, simultaneous protocol that measures all three within the same imaging session has, to our knowledge, not yet been established.

Hybrid PET/MRI provides a translational platform to combine established hypoxia tracers such as [^18^F]Fluoromisonidazole ([^18^F]FMISO) [19] with emerging molecular magnetic resonance imaging (MRI) methods. Hyperpolarized ^13^C MRI is a novel technique that has been translated into human studies [20], enables radiation‐free, non‐invasive assessment of metabolic flux [21], and has also enabled pH‐sensitive readouts using suitable probes [22, 23, 24, 25]. Integration of positron emission tomography (PET) with hyperpolarized ^13^C MRI has been demonstrated in preclinical small [26, 27, 28] and large animal studies [29, 30, 31, 32], and early clinical studies are emerging [33, 34]. To date, however, most combined PET–hyperpolarized ^13^C studies have focused on complementary assessment of tumor metabolism using hyperpolarized [1‐^13^C]pyruvate together with [^18^F]FDG PET, without directly addressing hypoxia or pH.

A method for robust, non‐invasive pH imaging is an unmet clinical need. Despite substantial efforts to characterize acidic tumor regions, a method to measure tissue extracellular pH is not routinely available in clinical practice and research, limiting testing of acidification models and the evaluation of pH as a biomarker for stratification or therapy monitoring [24, 35, 36, 37]. In addition, tumor heterogeneity renders imaging of the tumor microenvironment highly relevant: hypoxia, metabolism, and acidity can vary markedly within the same lesion, and sequential or separate measurements complicate co‐localization and interpretation.

**FIGURE 1:**
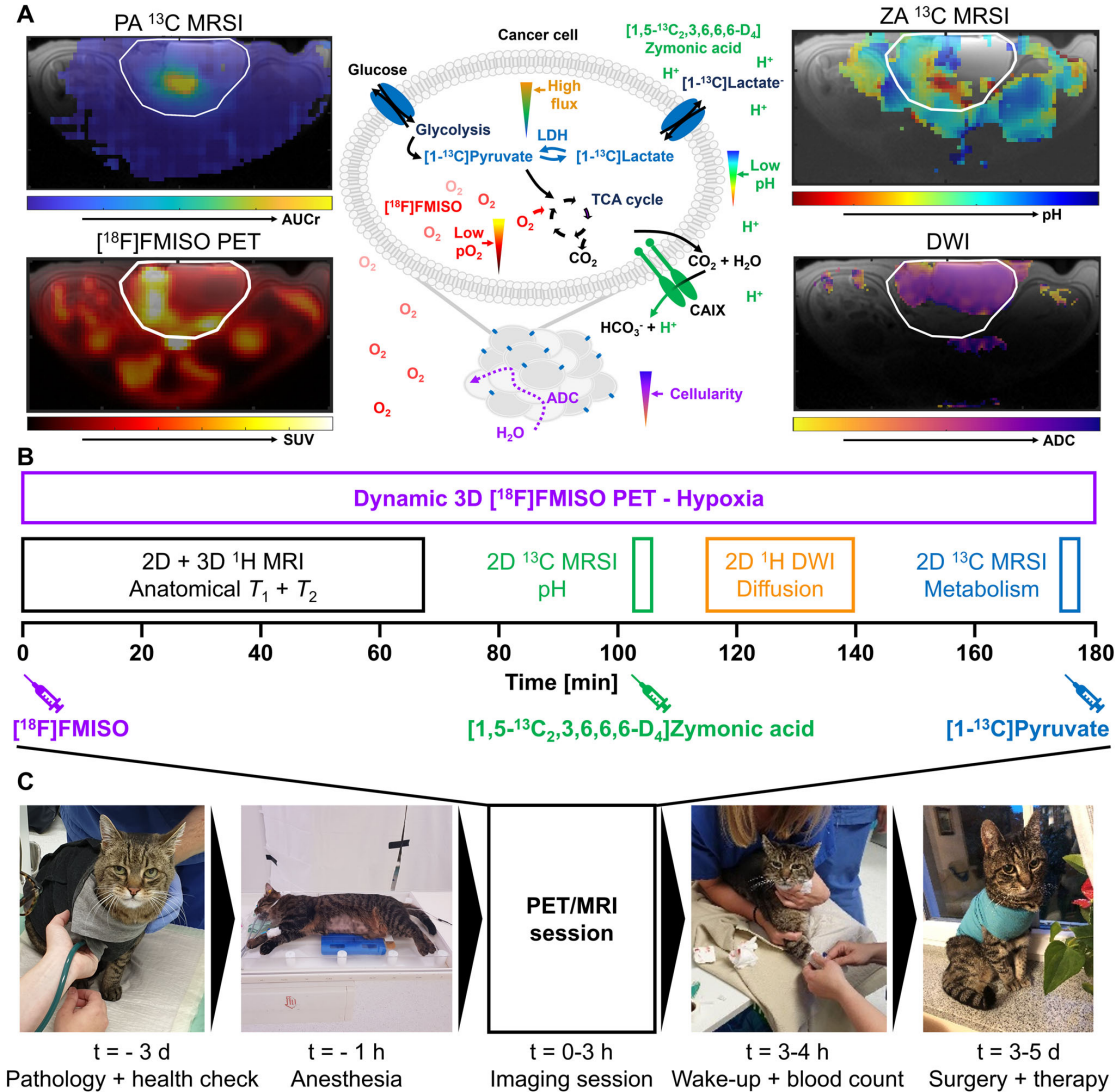
Study workflow to image key features of the tumor microenvironment—metabolism, hypoxia, pH, and cellularity. (A) Tumor cells often exhibit an increased demand for immediate energy, which is addressed by upregulation of glycolysis and, subsequently, cellular respiration. This can exacerbate hypoxia in dense tumor tissue of high cellularity. Additionally, an oxygen‐independent increased flux of pyruvate toward lactate is characteristic of cancer metabolic reprogramming. Metabolites such as lactate and carbon dioxide are then extravasated and due to co‐export of H^+^‐ions, acidify the extracellular space. Here, the conversion between carbon dioxide and bicarbonate is regulated by altered expression of carbonic anhydrases (e.g., CAIX). Hyperpolarized ^13^C MRSI using [1‐^13^C]pyruvate and [1,5‐^13^C_2_,3,6,6,6‐D_4_]zymonic acid, together with diffusion‐weighted imaging and [^18^F]FMISO PET, allows visualization of these key features in tumors (white region of interest (ROI)) in vivo. Exemplary parametric maps from different modalities are overlaid with *T*_2_‐weighted images, and absolute values for scale bars are omitted for schematic simplicity. (B) These modalities can be embedded in a multimodal imaging workflow starting with the injection of [^18^F]FMISO and a three‐hour‐long PET acquisition. MRI‐wise, anatomical imaging is followed by hyperpolarized ^13^C MRSI of zymonic acid and pyruvate, spaced by a diffusion‐weighted imaging (DWI) block. (C) This protocol is part of a comprehensive veterinary patient workflow, in which tumor diagnosis and a circulatory system check precede the imaging session, which starts with patient anesthesia. Side effects and patient comfort related to the agent injections are monitored during a wake‐up period, accompanied by blood counting. Lastly, tumors were resected surgically, and patients received appropriate follow‐up care, including post‐surgical wound care, pain medication, and infusion therapy. PA: Pyruvate, ZA: Zymonic acid.

Comparative oncology in domestic cats offers a particularly interesting path toward translation. Cats have basal metabolic rates closer to those of humans (approximately 2.5‐fold higher than the human rate, compared with ∼ 10‐fold higher in mice) [38, 39], and spontaneously arising feline sarcomas provide an abundant, clinically managed tumor model. These tumors arise in skin and connective tissues and are often linked historically to formerly used vaccines and their adjuvants (feline injection site sarcomas) [40]. Mesenchymal tumors are among the most frequent feline cancers and exhibit a high proportion of malignancy in a retrospective epidemiology study spanning over four decades [41]. Importantly, feline sarcomas share metabolic characteristics reported in human sarcoma, including increased glucose utilization and elevated pyruvate‐to‐lactate conversion [42]. This metabolic rewiring is often independent of hypoxia [2, 43], which itself is often present in solid, dense tumors with high cellularity. Subsequent extravasation of lactate can then cause acidification of the extracellular space, thereby promoting immune escape and proliferation (Figure 1A).

Within this work, we implement and evaluate a simultaneous imaging protocol on a clinical 3 T PET/MRI system that combines hyperpolarized ^13^C magnetic resonance spectroscopic imaging (MRSI) of pH using [1,5‐^13^C_2_,3,6,6,6‐D_4_]zymonic acid [22, 44], hyperpolarized ^13^C MRSI using [1‐^13^C]pyruvate to probe metabolism, and PET‐based hypoxia imaging using [^18^F]FMISO in feline sarcoma patients. We report the feasibility of a combined imaging approach that, for the first time, implements spatially resolved pH mapping in spontaneous sarcoma alongside coregistration with hypoxia and metabolic flux maps. We further explore spatial heterogeneity in these parameters both across tumors and within individual tumors. The presented protocol integrates the assessment of three frequently invoked tumor hallmarks in a clinically relevant setting, thereby supporting the mechanistic evaluation of the relation between hypoxia, metabolism, and pH and to inform translational development of pH‐sensitive imaging biomarkers for diagnosis, stratification, and therapy monitoring.

## 2 Results

### 2.1 Tumor Morphology

**FIGURE 2:**
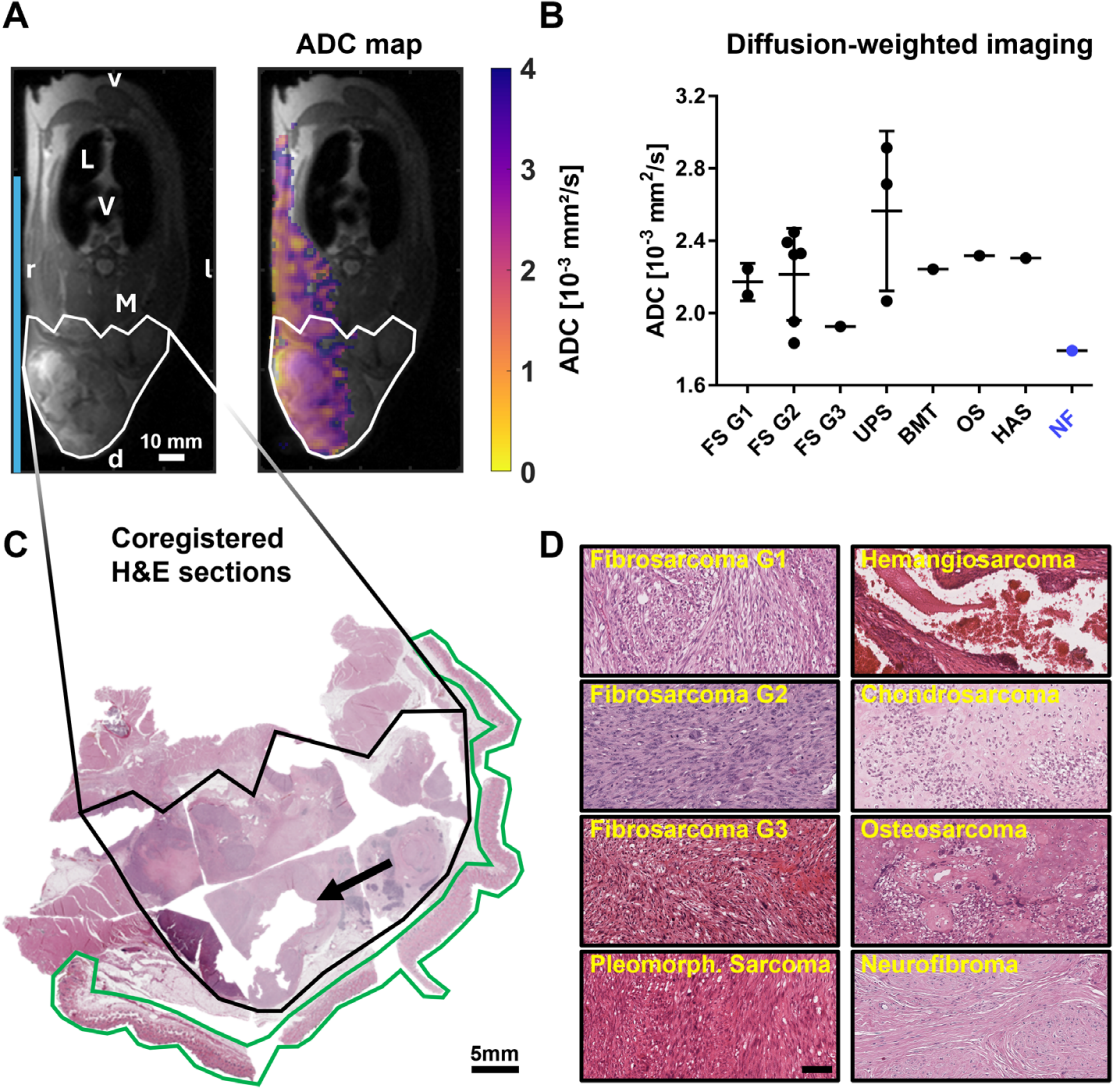
Tumor characterization by DWI and histopathology. (A) Left: Axial anatomical *T*_2_‐weighted image of the chest region of a cat lying on its side on a ^1^H/^13^C Tx/Rx surface coil (blue stripe). Additional orientation markers are r: right; l: left; v: ventral; d: dorsal; L: lung; M: muscle; V: central vessels (aorta, vena cava); white ROI: tumor. Right: ADC map overlaid with left anatomical image. The exemplary tumor (FS G2) shows margins of low diffusivity and a core with higher diffusivity. (B) ADC values grouped by tumor subtype. (C) Alignment of H&E tumor patches with MR image in (A). Tumor margins and skin are indicated by black and green contours, respectively. The black arrow indicates a cavitation within strongly heterogeneous tumor tissue. (D) Exemplary areas (20x) of H&E sections displaying cellular characteristics of the respective tumor type, scale bar 100 µm.

In total, 17 patients (Table S1) with biopsy‐confirmed malignant mesenchymal neoplasms and one benign neurofibroma patient were included in the study and underwent the full imaging protocol, consisting of hyperpolarized ^13^C MRSI, PET, and anatomical imaging (Figure 1B,C). All monitoring parameters acquired during the imaging session show no adverse trends and indicate that all cats tolerated the injections and anesthesia well (Figure S1). Relative comparison of pre‐ and post‐imaging blood counts and serum analysis, as well as comparison to reference ranges, shows no relevant adverse hematologic effects due to the imaging‐related protocol and injections (Figure S2). All lesions manifested as identifiable but not always clearly delimitable in both *T*_1_‐ and *T*_2_‐weighted images. No additional secondary lesions or metastasis were observable by MRI. Several tumors showed anatomical heterogeneity consisting of areas of solid tumor tissue and areas of tissue‐loss (cysts, necrosis), including free fluid. Accordingly, diffusion‐weighted imaging (DWI) revealed regions of high and moderately low apparent diffusion coefficient (ADC) values for fluid‐containing and solid tissue areas, respectively (Figure 2A). Whole‐tumor ADC values appear relatively high but are of a similar range for all imaged tumors (Figure 2B). Coregistration of hematoxylin & eosin (H&E) histomorphology of tumor sections was achieved using tissue‐inked tumor margins and a standardized trimming protocol. Stainings confirm tissue heterogeneity and cavitations, potentially due to cysts or largely necrotic areas (Figure 2C).

Detailed analysis of all neoplasms by experienced veterinary and human pathologists allowed to classify the tumors in this study into two fibrosarcomas (FSs) grade (G)1, seven FS G2, one FS G3, three undifferentiated pleomorphic sarcomas (UPSs), shortly termed pleomorphic sarcomas, one hemangiosarcoma (HAS), one chondrosarcoma (CS), one osteosarcoma (OS), and one “bimorphic tumor” (BMT). For the latter, two distinct morphological growth pattern (GP) compartments were observable, but no precise subtype classification based on H&E and immunohistochemistry was possible due to the lack of feline tissue specific markers. One tumor resection specimen was finally classified as a benign neurofibroma (NF) according to the veterinary WHO classification criteria [45]. Exemplary images of subtype‐specific tumor morphology are summarized in Figure 2D. Notably, all malignant tumors exhibit relatively high ADC values and show no difference between FS grade, while the benign NF reveals the lowest ADC.

### 2.2 Tumor Acidification

**FIGURE 3:**
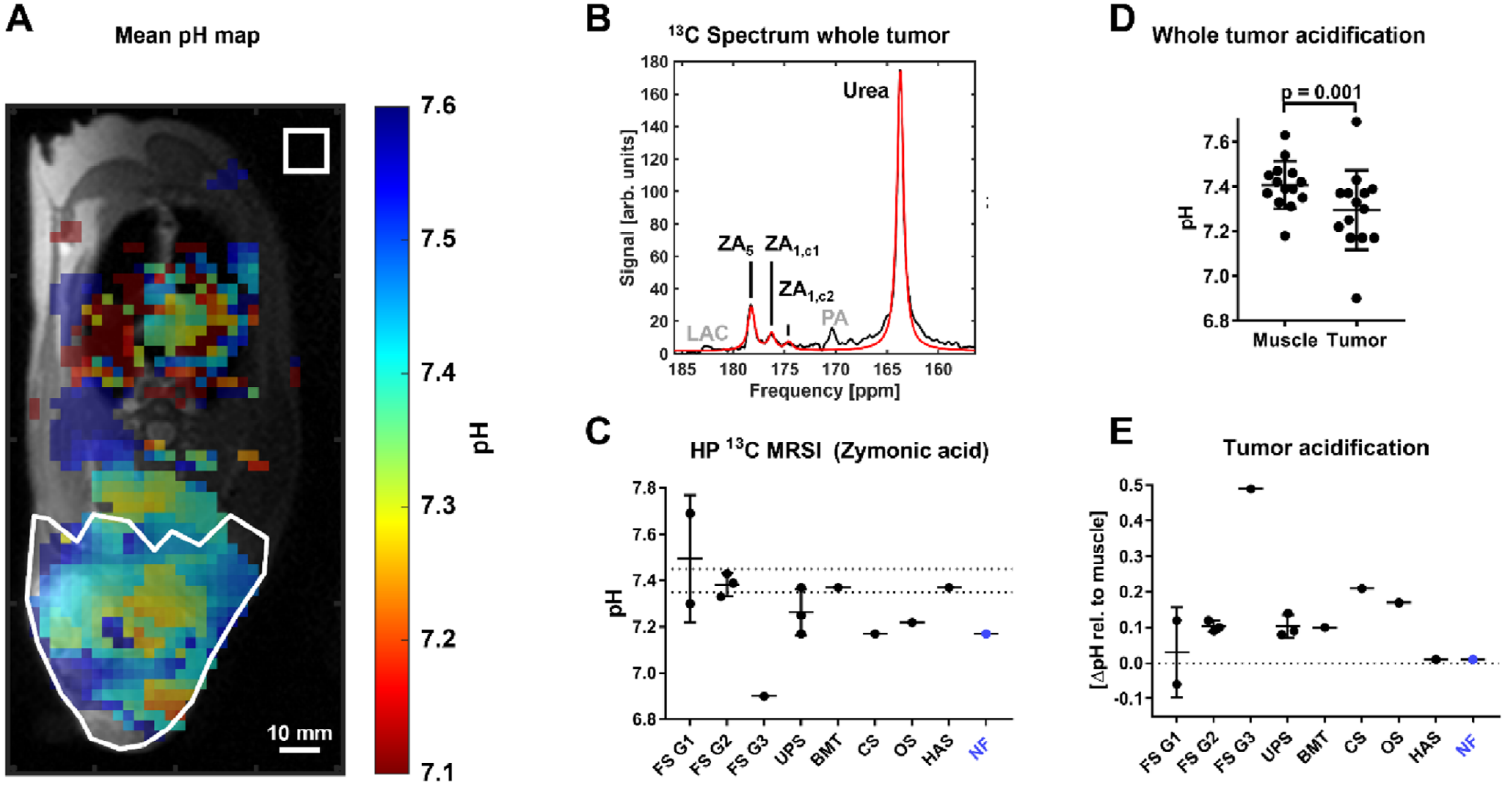
pH imaging in vivo reveals tumor acidification. (A) The mean pH map reconstructed from MRSI acquisitions on hyperpolarized [1,5‐^13^C_2_,3,6,6,6‐D_4_]zymonic acid and [^13^C]urea within tumors (exemplary FS G2, white ROI) often exhibited one or multiple acidified hotspots. The white square indicates the native MRSI resolution. (B) ^13^C spectrum averaged across the tumor in (A). Depending on signal intensity and ^13^C‐label, one (ZA_1,c1_) or two compartments (ZA_1,c2_) could be fitted relative to urea as a reference. Additional peaks are assumed to correspond to pyruvate (PA) and lactate (LAC) from partial decomposition of zymonic acid into pyruvate and subsequent metabolism. (C) Several tumor types exhibit pH values below the physiological range. FSs G2 show high consistency for three subjects. The physiological pH range (7.35–7.45) is indicated by dashed lines, and acidification is defined relative to muscle tissue. (D) Imaged tumors exhibit overall acidification compared to healthy muscle tissue of the same subject. (E) pH referencing of the tumor relative to muscle tissue reveals increasing acidification with higher FS degree. The CS and OS show relatively strong acidification, while low‐grade FSs and the benign NF (marked with blue color) show no acidification. A paired *t*‐test was used in (D).

pH imaging using [1,5‐^13^C_2_,3,6,6,6‐D_4_]zymonic acid provided sufficient signal strength, which allowed pH to be mapped in the majority of all imaged tumors with high spatial coverage. This reveals acidified hotspots (Figure 3A), where the mean pH is acidified by up to 0.3 pH units, while most of the tumor area has a physiological pH. These acidified hotspots manifest themselves as acidified pH compartments in the tumor, detectable as an additional spectral peak for the C_1_‐resonance of zymonic acid (Figure 3B).

The corresponding C_5_‐peak was often not visible due to the lower pH sensitivity and decreased spectral resolution at 3 T compared to higher fields [22]. The additional pH compartment is assigned to the extracellular space [22], while the first compartment originates from tumor vessels. A comparison of absolute pH values of the different tumor groups revealed a decreasing pH with increasing FS grade and tumor pH values being up to 0.2 pH units below the physiological range for many imaged tumors, including the benign NF (Figure 3C). The most aggressive FS G3 exhibits a mean pH below 7.0. In addition, a comparison of tumor pH to healthy tissue pH from the same animals also revealed tumor acidification (pH 7.30 ± 0.18) compared to muscle pH (paired *t*‐test, pH 7.41 ± 0.11, *p* = 0.001, Figure 3D). However, the muscle pH values also vary strongly between subjects, and systemic body pH is known to be prone to isoflurane anesthesia [23]. To remove intersubject variations, which can depend on daily animal condition and variations in anesthesia conditions, tumor pH values were subtracted from the respective muscle pH from the same animal to calculate the effective tumor acidification (Ac.). Here, tumor‐adjacent muscle is chosen as the tissue pH reference, as pH values agree with pH values from blood vessel regions (Figure S3), but muscle regions are often closer to the coil and exhibit better signal‐to‐noise ratios. Here, FSs of different grades can show acidification of up to 0.5 pH units (Figure 3E), with a trend to higher acidification for higher FS grade. Bone‐derived sarcomas (CS, OS) also show pronounced acidification, which aligns with previously reported findings [46]. Notably, the HAS and the benign NF show no acidification, potentially either due to strong buffering by blood or non‐malignancy.

### 2.3 Tumor Metabolism

**FIGURE 4:**
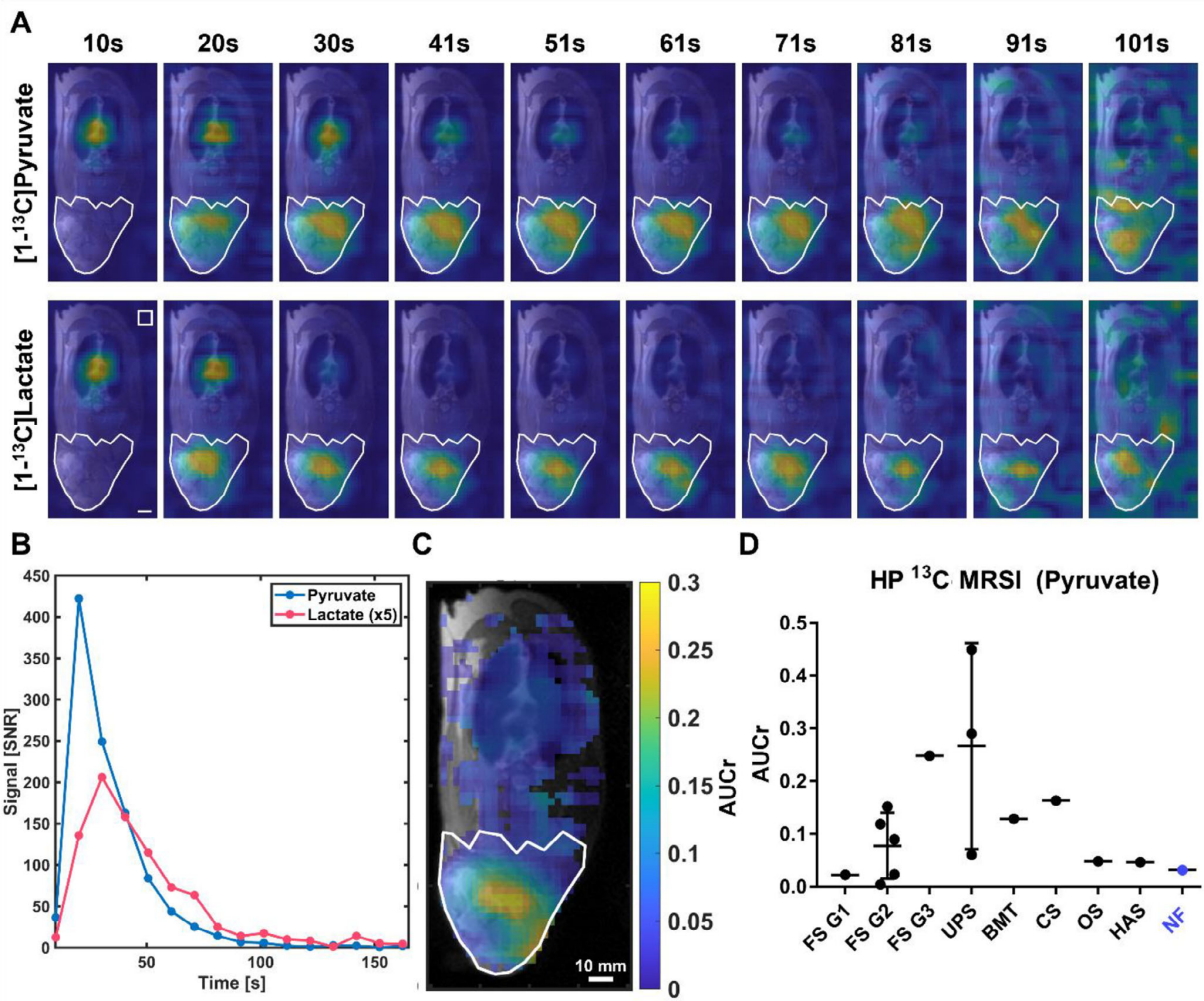
Imaging tumor metabolism in vivo. (A) Image time series of [1‐^13^C]pyruvate and [1‐^13^C]lactate. The FS G2 shown as an example reveals regions of high perfusion (strong pyruvate signal) and pronounced metabolism (strong lactate signal). The white ROI and square indicate the tumor and the native MRSI resolution, respectively, scale bar 10 mm. (B) Whole‐tumor time curve showing the pyruvate bolus and labeling of lactate. (C) AUCr image calculated from the time series in (A) reveals a metabolic hotspot. (D) Whole‐tumor AUCr values vary between FS grade and showed substantial variation among UPSs.

To probe altered metabolism, hyperpolarized ^13^C MRSI of [1‐^13^C]pyruvate and subsequent exchange with [1‐^13^C]lactate was performed. Time series of pyruvate (top) and lactate (bottom) images showing the first ten frames from a dynamic MRSI acquisition started with a bright bolus in a central blood vessel, followed by strong pyruvate and lactate signal in a central tumor region (Figure 4A). Whole‐tumor time curves (Figure 4B) show a strong pyruvate bolus and corresponding ^13^C‐labeling of lactate. Using these pyruvate‐ and lactate time curves to calculate area‐under‐the‐curve ratio (AUCr) maps on a voxelwise basis identifies hotspots of high metabolic flux within the tumor region (Figure 4C). Interestingly, qualitative comparison to the pH map in (Figure 3A) shows a similar localization of acidified and metabolically active hotspots. Comparing AUCr values on a whole‐tumor basis (Figure 4D) reveals differences between subtypes similar to the findings from pH imaging and potentially higher metabolic activity for higher FS grade.

UPSs show the highest overall AUCr values but also strong intersubtype variation. The CS turns out to have a markedly higher AUCr value compared to the OS. Similar to the acidification pattern, the HAS and NF show only low metabolic activity relative to the other subtypes.

### 2.4 Tumor Hypoxia

**FIGURE 5:**
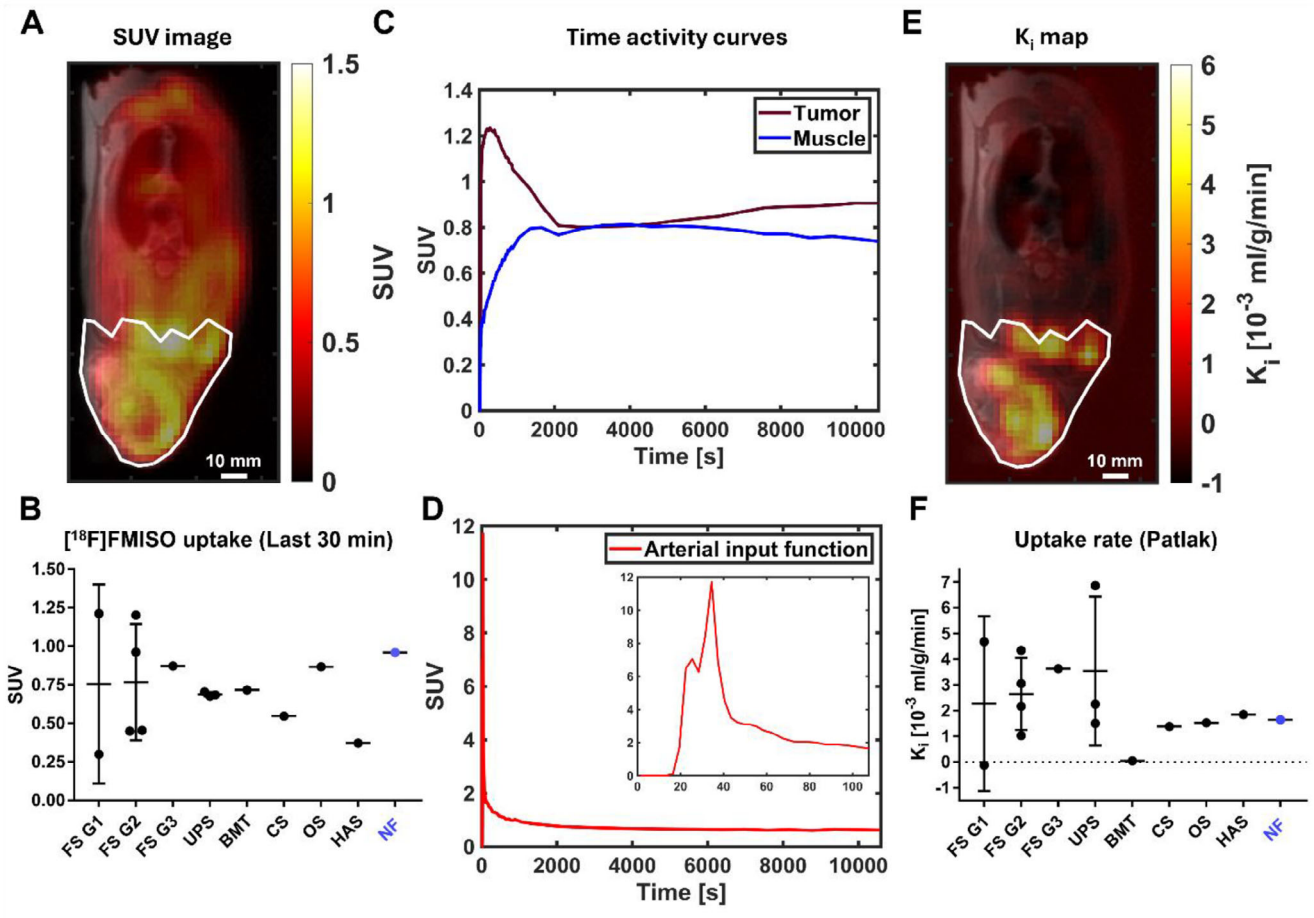
Tumor hypoxia visualized by the kinetics of [^18^F]FMISO PET. (A) Exemplary SUV image of a FS G2 summed over the last 30 min from the three‐hour acquisition. (B) Mean tumor SUV values summed over the last 30 min showed strong variation within and between tumor groups. Notably, the NF exhibits a high [^18^F]FMISO uptake. (C) Time activity curve for the tumor in (A) and a healthy spine muscle region of the same patient. (D) For kinetic modeling, a time activity curve from a central blood vessel in proximity to the tumor was selected as an arterial input function. Inset: Magnification of the first 100‐s interval. (E) The K_i_ map corresponding to (A) shows better tumor contrast relative to the SUV image. (F) The whole‐tumor pattern for FS is similar to observations from SUV, while variation among UPSs strongly increases.

To investigate the presence of hypoxia in the sarcoma cat patient cohort as a complementary marker to pH and metabolism, [^18^F]FMISO PET was quantified by two well‐established parameters, namely standard uptake value (SUV) and Patlak‐derived K_i_. The former is obtained by summing the last 30 min from the PET acquisition and provides a measure similar to late time point static imaging in clinical settings. The resulting SUV images (Figure 5A) show strong, heterogeneous uptake in the tumors; however, with low contrast compared to directly adjacent muscle tissue. Comparison of the static uptake between tumor subtypes shows substantial variation within the FS groups (Figure 5B). Static uptake in the other subgroups also varies strongly between groups, with the benign NF belonging to the most hypoxic entities. In contrast, UPSs show strong consistency in SUVs for three tumors. The low contrast between muscle and tumor tissue might be explained by the slow washout kinetics of [^18^F]FMISO. This is indicated by the time activity curves of the tumor and a muscle region (Figure 5C). Here, the kinetics of both tissue types can be clearly distinguished but are not strongly reflected by absolute uptake values within the imaging window. To imprint the kinetic variation into the image contrast, image‐derived arterial input functions (Figure 5D) were used for voxelwise Patlak modeling of the time activity curves.

The resulting parametric K_i_ maps show several hotspots of similar location as for the static SUV images; however, with increased contrast compared to the static images (Figure 5E). Interestingly, differences in hypoxia found from SUV for CS, OS, HAS, and NF are not reflected in K_i_ (Figure 5F). While relative differences between FS grades remain analogous to SUV, the K_i_ varies stronger within the UPS group compared to SUV. The benign NF shows a high SUV, but only moderate K_i_ relative to all other subtypes. Validation of hypoxia detection was assessed using pimonidazole, quantified as positive cell fraction on central immunohistochemical sections. SUV shows a slightly stronger, but not significant correlation with pimonidazole positive cell fraction (Figure S4). Overall, all tumors show pronounced hypoxia, exceeding 40% positive cell fraction in all cases.

### 2.5 Intertumoral Heterogeneity

**FIGURE 6:**
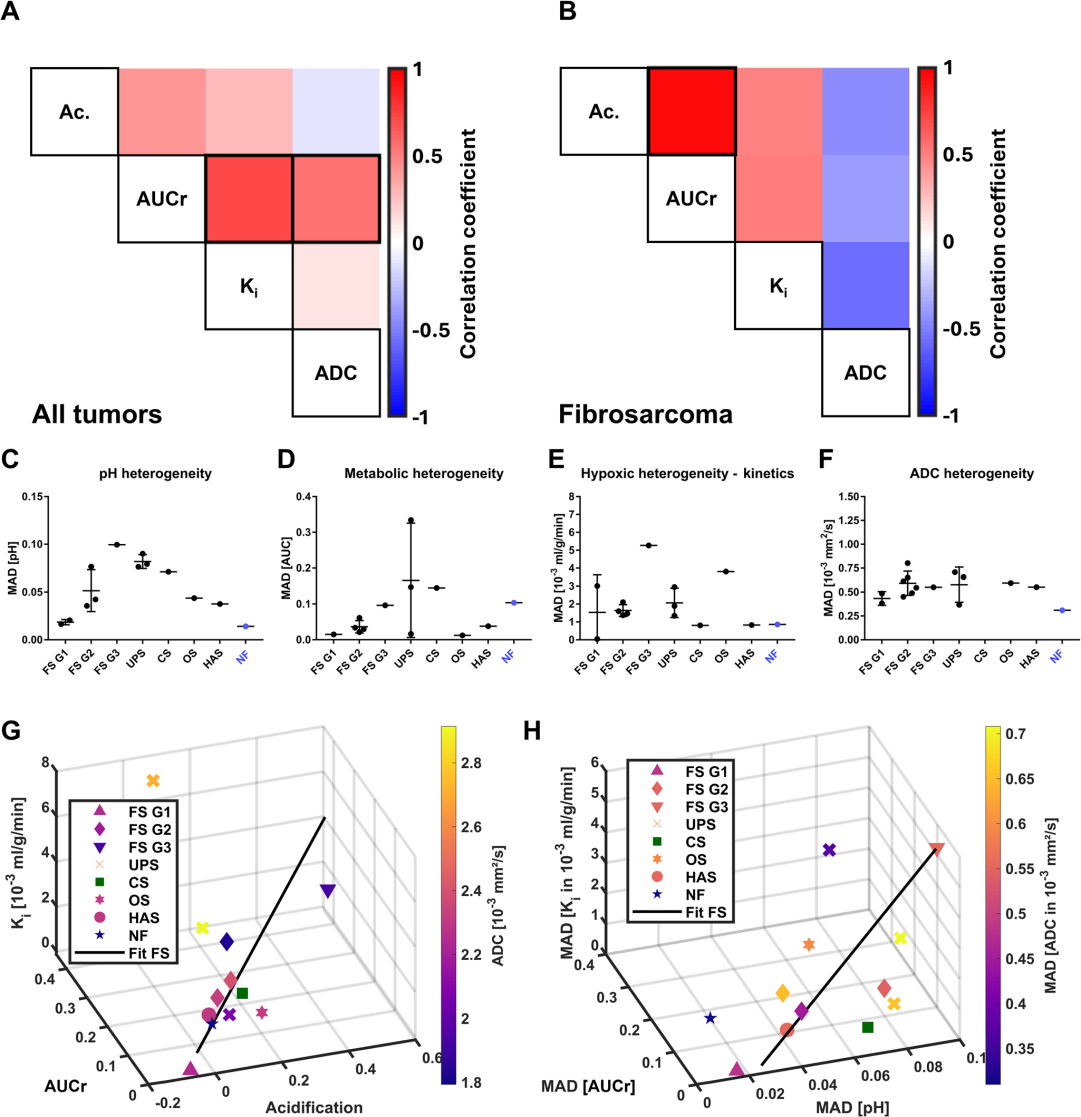
Intertumoral correlation of metabolic characteristics. Pairwise correlation matrix for six imaging parameters for all tumor types (A) and only FS of different grade (B). Statistically significant correlations (*p <* 0.05) are framed with black squares. The heterogeneity, measured by the MAD of pH (C) and metabolism (D) increases strongly with FS grade. In UPSs, pH heterogeneity is more consistent than metabolic heterogeneity. (E) Hypoxic and (F) ADC heterogeneity show no pattern and vary strongly within and between tumor groups. (G) Visualization of individual tumors as 4D feature coordinates indicates the majority of the tumors to be located along a trajectory in the 3D metabolic subspace, represented by a vector from PCA on all FS. (H) Visualization of heterogeneity coordinates with a similar trend as in (G). An animated version of (G) and (H) can be found in Animation S8 and S9, respectively.

Following the abovementioned 1D subtype comparisons of just one single parameter, a better understanding of the metabolic processes in sarcomas can be gained by investigating how two or more parameters might be connected to each other. Due to sample size constraints, correlations between imaging biomarkers were only assessed for all tumors and the FS subgroup. Four imaging biomarkers derived from the multimodal imaging protocol (acidification, AUCr, K_i_ and ADC) were assessed pairwise for correlations. The results are summarized in the correlation matrices involving all tumor subtypes (Figure 6A) and the FS subtypes of different grades (Figure 6B). The matrix involving all subtypes shows a strongly positive correlation (*r* = 0.71, *p* = 0.006) between AUCr and K_i_, suggesting that metabolic flux from pyruvate to lactate is a result of the low availability of oxygen, potentially reducing the shuttling of pyruvate into the TCA cycle. A similar correlation is found for FSs only, which is not significant (*r* = 0.50, *p* = 0.39). Across all subtypes, AUCr also correlates weakly positively with ADC (*r* = 0.55, *p* = 0.05), which is opposite for FS (*r* = −0.39, *p* = 0.45), and due to two UPS entities of particularly high ADC and AUCr, a respective example is shown in Figure 1A. On the other hand, FSs of varying grade show a strongly positive correlation between lactate labeling and acidification (*r* = 0.89, *p* = 0.04), which is even more pronounced for absolute pH and lactate production (*r* = −0.97, *p* = 0.008, not shown). The same trend is observed when taking into account all other subtypes, but no significance is reached. Overall, the correlation pattern for FSs as a subtype group and all subtypes is similar. In addition to the absolute mean values, tumor heterogeneity with respect to the examined features can be compared using the mean absolute deviation (MAD). This value reflects the variation across all valid voxels within the tumor for each modality, as tumor heterogeneity cannot be assumed to follow a Gaussian distribution. Here, pH heterogeneity follows a trend similar to absolute acidification, where the most aggressive tumors (FS G3 and UPSs [47]) show the highest heterogeneity with good consistency within subgroups and low heterogeneity for low‐grade and benign tumors (Figure 6C). While a similar trend is observed for metabolic heterogeneity (MAD of AUCr, Figure 6D), variation among UPSs and heterogeneity in the benign NF are increased. Similarly, hypoxic heterogeneity (Figure 6E) also appears to increase with FS grade, which is not seen for ADC heterogeneity (Figure 6F). Notably, some subtypes seem to show comparably high heterogeneity for just one parameter (hypoxia in OS) while others show comparably high heterogeneity for all parameters (e.g., FS G3). Additionally, all parameters (acidification, AUCr, K_i_ and ADC) can be combined into a 4D feature coordinate. This coordinate can be plotted in a 3D ’metabolic space’ (Figure 6G) where the ADC is color‐coded (CS without valid ADC measurement). Principle component analysis (PCA) on the three spatial dimensions returns an Eigenvector, which suggests that hypoxia, AUCr, and acidification are connected to each other, thereby supporting the suggested functional tumor model (Figure 1A). However, the extent appears to depend on the aggressiveness for the majority of sarcomas, with UPSs deviating most strongly from this finding. A similar pattern can be observed when plotting the MADs in an analogous manner (Figure 6H). While heterogeneity of different metabolic features appears to be linked to each other, tumors of higher aggressiveness, particularly FS, appear at the upper end of this metabolic trajectory, pointing toward sheterogeneity as a potential marker for tumor aggressiveness in FSs.

### 2.6 Intratumoral Heterogeneity

**FIGURE 7:**
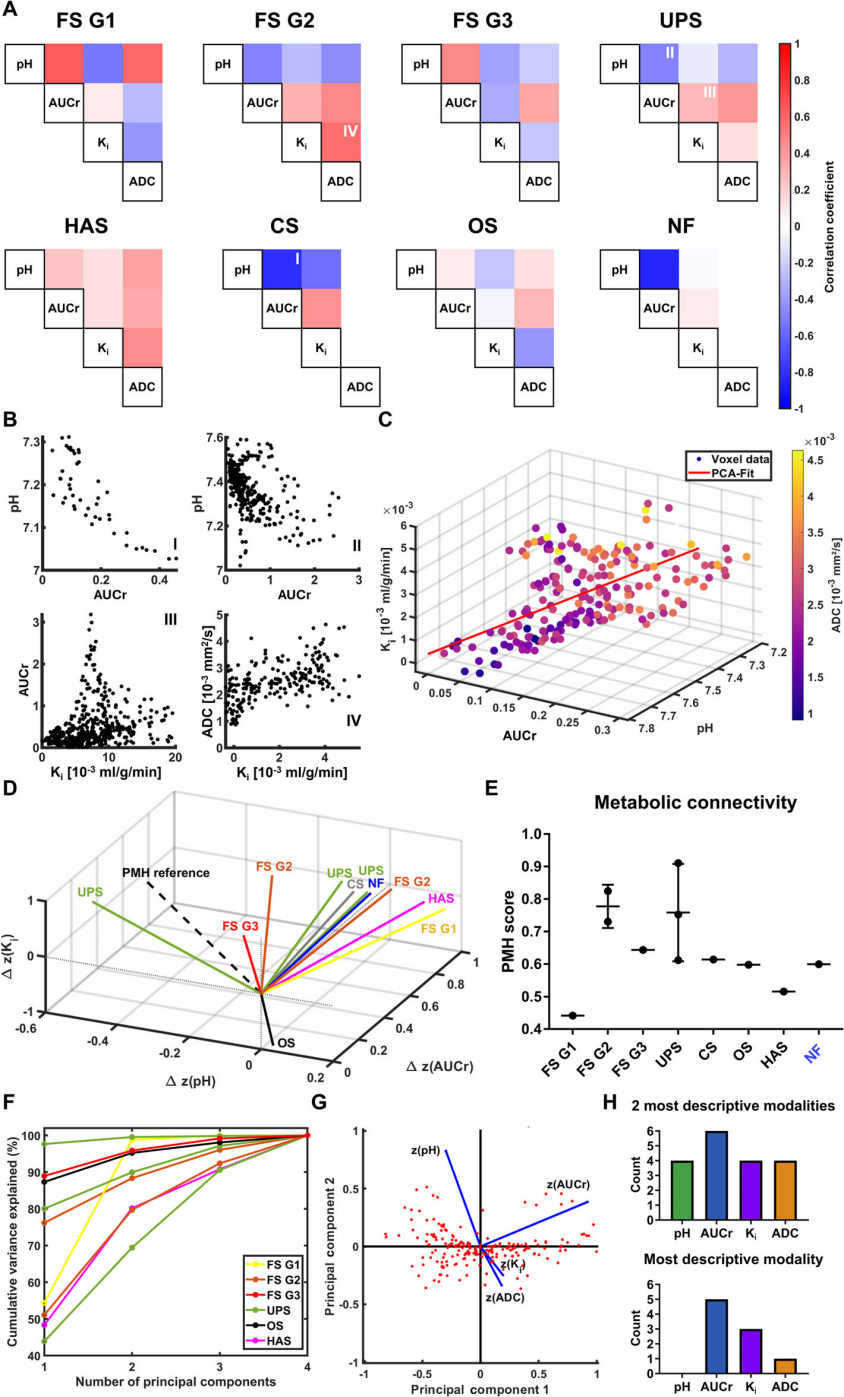
Intratumoral correlation of metabolic characteristics. (A) Representative correlation matrices for all tumor types and grades within this study. ADC data for the CS and the NF did not allow voxel‐based correlation analysis. (B) Selected correlations from matrices in (A) are indicated with Roman numerals. (C) Visualization of tumor voxel parameters from hyperpolarized ^13^C MRSI and PET as metabolic coordinates for an FS G2. The red line indicates a PCA vector derived from the three spatially visualized parameters. (D) 3D plot of normalized ’glycolytic’ vectors derived from PCA on pH, metabolism, and hypoxia from PCA analysis as performed in (C). (E) PMH scores are highest for FS G2, FS G3, and UPS compared to other tumors within the study cohort. (F) Cumulative variance for all tumors with complete dataset, indicating that most tumors are well described by two components. (G) The biplot for the same subject as in (C) indicates pH and AUCr as the strongest loadings for principal components 1 and 2. (H) Subset analysis describing how often each of the two modalities belongs to the two most intratumoral heterogeneity‐capturing modalities (top) or a single modality (bottom) reveals AUCr as most descriptive for intratumoral heterogeneity. An animated version of (C) and (D) can be found in Animation S10 and S11, respectively.

While intertumor correlations provide insight into differences between sarcoma subtypes, intratumoral correlations of parameters provide insight into the metabolic relationships within a single tumor or across the full population subset. For this purpose, imaging parameters were correlated on a voxelwise basis to yield tumor‐specific correlation matrices. An overview of example matrices for each subtype is shown in Figure 7A. Here, patterns show huge variations between subtypes and even among subtypes of varying grades. Nevertheless, it should be noted that analogous to the intertumor correlation, several subtypes show a correlation between pH and AUCr (Figure 7B I, II), which further supports the assumption of a lactate‐driven acidification mechanism. Within the same UPS, lactate metabolism correlates with hypoxia (Figure 7B III). The distribution further indicates that lactate labeling increases in hypoxic areas. However, this observation alone does not establish hypoxia as the cause of the increase in this tumor. Interestingly, some tumors also show unexpected findings. For example, a FS G2 reveals a positive correlation between ADC and K_i_, which supports previous findings [48], and counteracts the assumption that dense tumor tissue promotes hypoxia (Figure 7B IV). To further increase insight into metabolic dependencies from the applied imaging modalities, all parameters for each tumor voxel can be plotted as 4D feature coordinates analogous to Figure 6G. Exemplary for a FS G2 (Figure 7C), the tumor voxel distribution aligns along a trajectory in a 3D subspace, which can be visualized by a PCA‐derived trajectory (red). This suggests a mechanistic relation between hypoxia, subsequently altered metabolism, and resulting extracellular acidification. Further, higher ADC values appear to be related to lower pH, higher AUCr, and increased K_i_. To facilitate cross‐tumor comparison, PCA was repeated using z‐scores for all parameters, thereby mitigating scale effects and enabling direct assessment of parameter dependencies across tumors. Plotting the corresponding unit vectors, analogous to Figure 7C, yields a distribution of ‘metabolic vectors’ that characterizes the relationships between the metabolic parameters within each tumor (Figure 7D). Weakly aggressive tumors (FS G1, NF) show little or no acidification potential (Δz(pH) ∼ 0 or Δz(pH) < 0) in contrast to many aggressive tumors (FS G2, FS G3, UPS). The extent to which each tumor relies on anaerobic glycolysis for extracellular acidification is quantified by the PMH score (Experimental Section ‘Statistics and Principal Component Analysis’; Figure 7E). Consistently, FS G2, FS G3, and UPS, which are among the most aggressive tumors in our cohort, exhibit the highest scores compared to low grade (FS G1) or benign tumors (NF). Despite the acquisition of four imaging biomarkers, the cumulative explained‐variance plot suggests that the heterogeneity of most tumors can be described by only two principal components, which account for more than 80% of the variance in almost all tumors (Figure 7F). An exemplary biplot (Figure 7G; same subject as in Figure 7C), visualizing the relationships between principal components and measured parameters, shows that pH and AUCr derived from hyperpolarized ^13^C MRSI contribute most strongly to the observed heterogeneity. From a practical viewpoint, it would be interesting to reduce the number of imaging modalities while still assessing intratumoral heterogeneity in its full complexity. From subset‐based PCA analysis determining the two most heterogeneity‐capturing modalities, all four modalities appear to be of similar importance when assessing how often each modality belongs to the two modalities that describe most of the heterogeneity within one tumor entity (Figure 7H top). Clinically, instead of employing two imaging modalities, it is even more of interest to identify the single most informative modality if acquisition had to be limited to one. Based on a second corresponding subset analysis, AUCr derived from hyperpolarized ^13^C MRSI of [1‐^13^C]pyruvate can be identified as the most informative modality (Figure 7H bottom).

### 2.7 Patient Case ‐ Bimorphic Tumor

**FIGURE 8:**
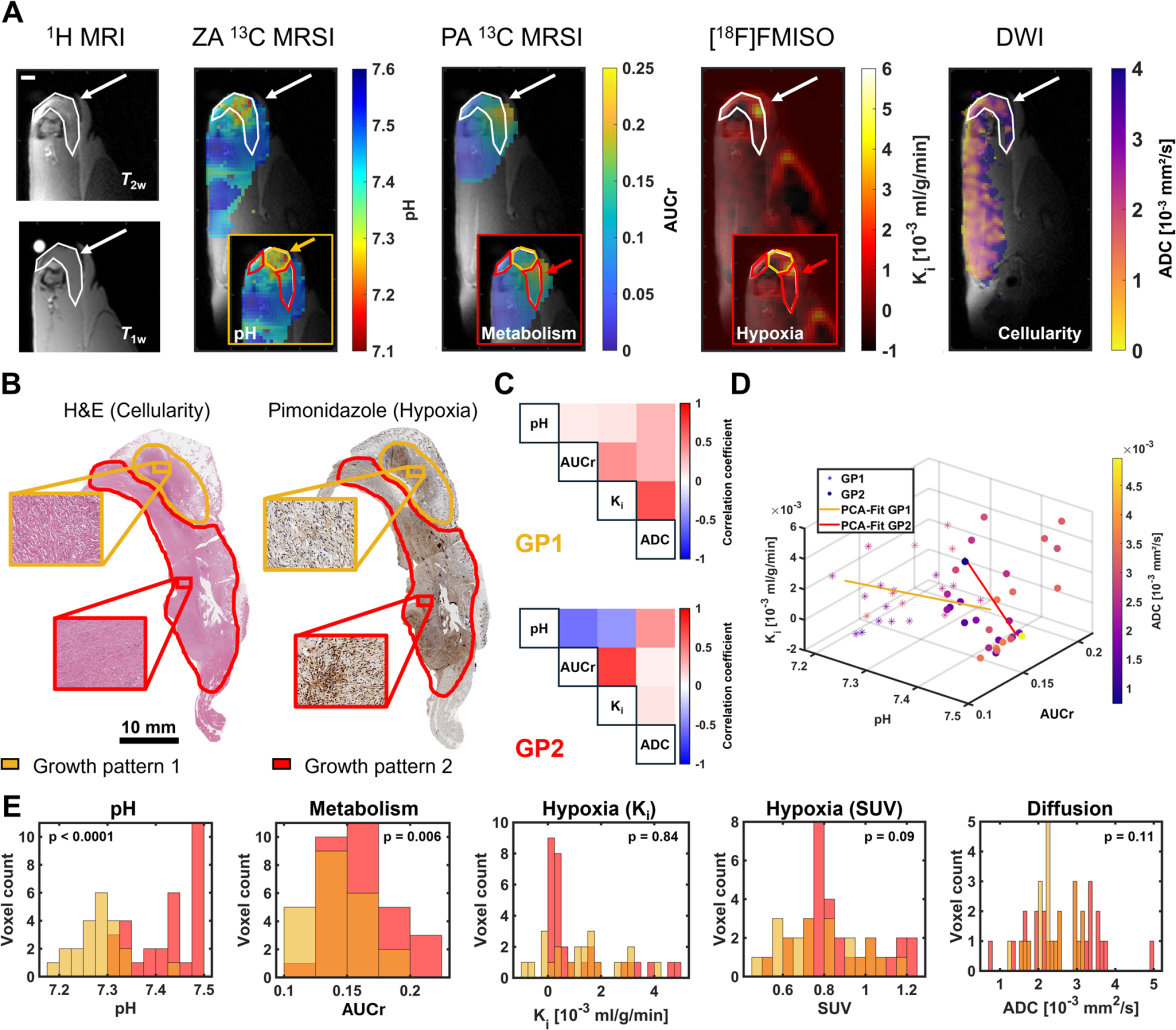
Multimodal imaging reflects tumor compartmentation in a bimorphic tumor. (A) In contrast to anatomical images, parameter maps from hyperpolarized ^13^C MRSI and PET reveal two distinct metabolic hotspots, where pH is complementary to AUCr and [^18^F]FMISO uptake, scale bar 10 mm. The bright spot within the lesion in the *T*_2w_ image results from a surface coil flare. (B) Histopathology reveals a bimorphic tumor, which consists of two distinct morphologic GP (GP1: yellow; GP2: red) due to the inability of subtype assignment. Insets in b show zoomed (40x) cellular details from both compartments. 2D‐ (C) and 3D (D) assessment of parameter correlation reveals distinct correlation matrices and PCA vectors for both compartments, respectively. (E) Welch's test‐based histogram comparison of five imaging parameters indicates pH and metabolism to best distinguish both compartments. An animated version of (D) can be found in Animation S12.

A particular case in this study was the finding of a bimorphic tumor, which presents histopathologically with two distinct morphological GPs. In the respective patient, the tumor looped around the right hindlimb and presented with an acidified hotspot in the pH map, complementary to another, dislocated hotspot of high metabolic flux and strong [^18^F]FMISO uptake. In contrast, anatomical images and the ADC map were inconclusive (Figure 8A). Upon resection and histological assessment by H&E (Figure 8B left), the tumor entity turned out to consist of two histomorphologically different compartments with distinct cellular GP (GP1 and GP2). However, neither H&E nor various applied immunohistochemical markers (section ‘Tumor Morphology’) allowed the unambiguous assignment of these GPs to a distinct sarcoma subtype. Staining for pimonidazole shows a slightly increased positive tissue fraction for GP2 (Figure 8B right) in line with strong PET uptake. Coregistration and ROI transfer from histology to the imaging modalities indicate the distinct hotspots to correspond to the different tumor compartments (Figure 8A insets). Intratumor correlation analysis analogous to Figure 7A clearly distinguishes the GP1 from the GP2 compartment (Figure 8C). Notably, the matrix pattern of the GP2 compartment matches overall findings for the FS subtype in Figure 6B and Figure 7A.

Further, both compartments show clearly distinguishable ’glycolytic vectors’, which indicates the possibility for non‐invasive differentiation of the compartments by multimodal imaging (Figure 8D). To assess the distinguishability of both compartments based on the applied imaging modalities, histograms of all voxels with assigned compartments were generated (Figure 8E). Here, pH allows a clear separation of both compartments (*p <* 0.0001) while metabolism also reliably differentiates between the compartments (*p* = 0.006). In contrast, PET parameters, namely K_i_ and SUV, as well as ADC, fail to distinguish the voxel populations, suggesting a high value for hyperpolarized ^13^C MRSI for characterization of intra‐ and intertumoral metabolic heterogeneity.

### 2.8 Prognostic Potential of Multimodal Imaging

**FIGURE 9:**
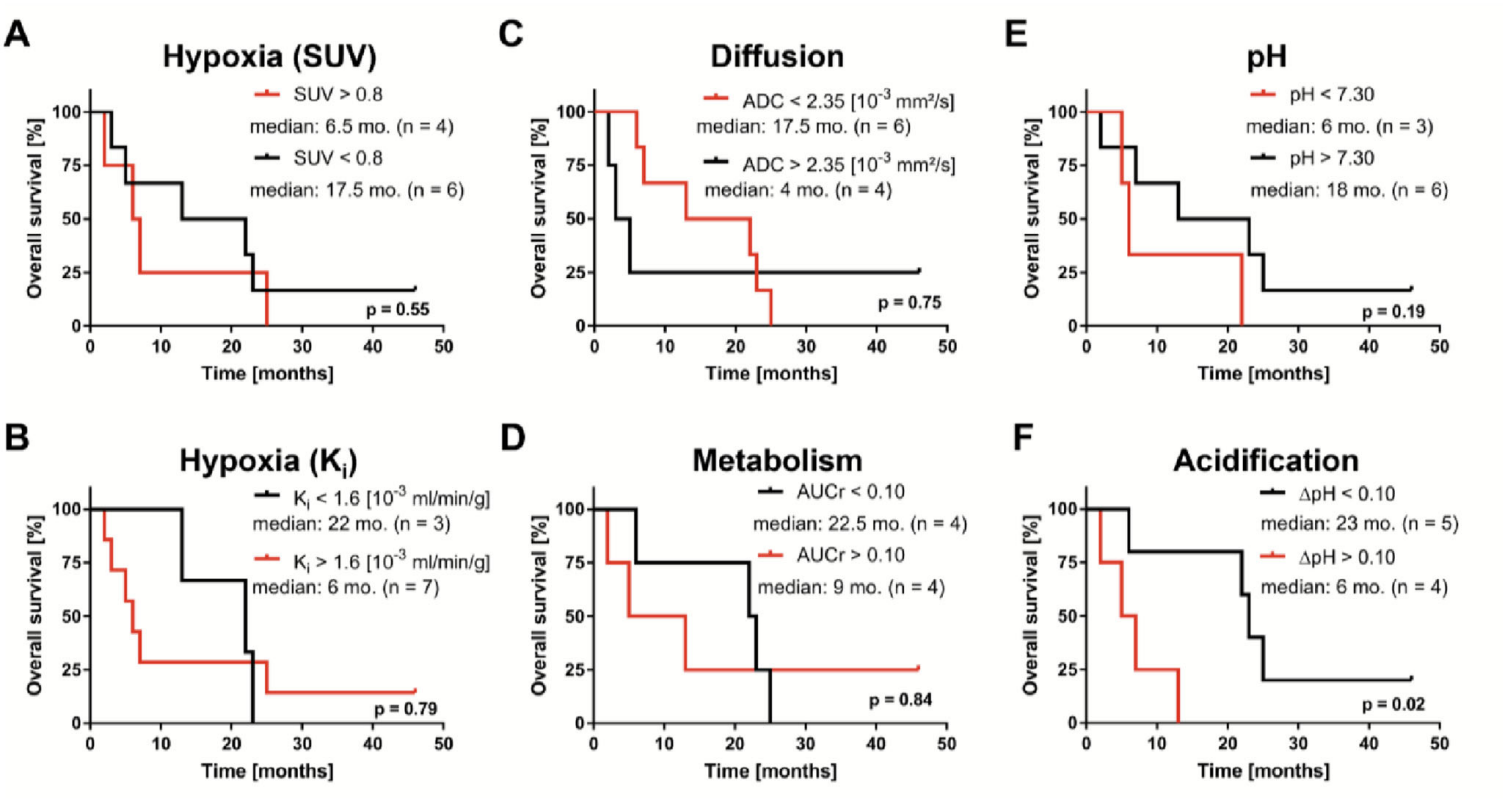
Kaplan–Meier survival analysis using imaging parameters. Survival curves from dichotomized groups for static (A) and kinetic (B) PET quantification, DWI analysis (C), and hyperpolarized ^13^C MRSI of metabolism (D) and pH (E, F).

The ability of each imaging biomarker to assess patient outcome was assessed by a follow‐up survey up to four years after study initiation (Figure S5). Stronger hypoxia, indicated by either increased SUV (Figure 9A) or K_i_‐values (Figure 9B) points toward reduced median survival, but is not conclusive. Interestingly, higher ADC values appear to be related to a more than four‐fold reduced median survival (Figure 9C), however without reaching significance. The same holds for hyperpolarized ^13^C MRSI, where higher metabolic flux of pyruvate to lactate (Figure 9D) shows a trend to a strongly reduced median survival.

For pH imaging, normalization of pH is important, as absolute pH (Figure 9E) does not allow for a clear distinction of patient groups. In contrast, acidification beyond 0.1 pH units is suggested to be a suitable cutoff, as it distinguishes a 3.8‐fold reduction of median survival (Figure 9F) and is therefore the most relevant prognostic imaging parameter within our small cohort study. Increased hypoxia, higher ADC, high metabolic activity, and strong acidification are unfavorable tumor characteristics as shown by group dichotomization. This is also reflected in a direct correlation analysis of overall survival duration and the respective parameters (Figure S6). In this case, acidification, metabolism and ADC exhibit the strongest correlations with overall survival, with the strongest acidified tumors resulting in the shortest survival times. Additionally, to assess potentially confounding factors, tumor volume, age, weight, sex, breed, and tumor location as meta parameters from Table S1 were assessed for their predictive potential (Figure S7A–F). None of them appears relevant to predict outcome of the cats within our cohort. Correlation of continuous meta parameters age, weight and tumor volume indicates a negative correlation of age with SUV and volume, while volume correlates positively with ADC and AUCr (Figure S7G). Categorical meta parameters show no relevant confounding influence on imaging parameters (Figure S7H), but interestingly, tumors located on the abdominal wall show a significantly lower acidification (mean ΔpH = 0.05) compared to tumors located on other parts of the body (mean ΔpH = 0.19, Figure S7H).

## 3 Discussion

This study set out to establish a clinically relevant workflow for simultaneous assessment of extracellular pH, metabolism, and hypoxia on a 3 T clinical PET/MRI system, using hyperpolarized ^13^C MRSI of pH alongside hyperpolarized [1‐^13^C]pyruvate MRSI and [^18^F]FMISO PET in spontaneous feline tumors. One of the challenges in the clinical translation of novel MRI techniques is moving from preclinical scanners, mostly operating at 7 T and beyond, to a clinical 3 T system. Here, spectral resolution is lower, which complicates spectral assignments, in particular for assessing pH. However, at 3 T compared to 7 T, there is also the benefit that ^13^C *T*_1_ relaxation time constants of pyruvate and zymonic acid are prolonged [44], which results in a longer availability of hyperpolarized magnetization and therefore better signal‐to‐noise ratio. Here, the technical feasibility, particularly the scaling of the hyperpolarization protocols to a clinical setting, and sound measurement accuracy could be demonstrated based on a comprehensive set of feline patient data. This clearly shows that chemical shift–based pH mapping using probes such as [1,5‐^13^C_2_,3,6,6,6‐D_4_]zymonic acid yields robust, spatially resolved readouts in a clinical setting. Together, these findings support the premise that hyperpolarized ^13^C pH imaging is not only technically viable but also holds value for further translational development.

Even though blood counting and animal monitoring indicated good tolerability of the injections and imaging procedures in this study, dedicated toxicology and development of sterile compounding workflows will be essential for clinical translation of [1,5‐^13^C_2_,3,6,6,6‐D_4_]zymonic acid. In contrast, [1‐^13^C]pyruvate has already been used in clinical trials [20], and [^18^F]FMISO is established in clinical practice at some centers, which lowers the barrier for future human implementation of the proposed combined protocol [19].

A key motivation for simultaneous, coregistered mapping of hypoxia, metabolism, and extracellular pH is tumor heterogeneity as these parameters can vary substantially within a lesion and sequential measurements complicate spatial correspondence and mechanistic interpretation. In this study, spontaneous feline tumors served as an intermediate model between rodents and humans, as they represent morphologic features of similar human neoplasms and are more comparable in both size and basal metabolic rate to human counterparts, which highlights the potential and applicability of spontaneously arising neoplasms in pets in comparative biomedical studies [49]. The high inter‐ and intratumoral variability observed within this study cohort emphasizes this point. At the same time, the modest cohort size and limited numbers per histologic subtype require cautious interpretation, particularly for claims about subtype‐specific biological mechanisms.

Interestingly, DWI resulted in comparably high ADC values (1.79–2.91 ∙ 10^−3^ mm^2^/s) for the entire study cohort. Similarly high values have been found previously [50] for benign and malignant soft tissue tumors, among which were sarcomas. However, lower values are often observed for malignant lesions (0.87–1.40 ∙ 10^−3^ mm^2^/s) [51] and, in particular, soft tissue sarcomas (0.47–2.09 ∙ 10^−3^ mm^2^/s) [52, 53, 54], with strong heterogeneity for the latter due to varying cellularity, extracellular matrix composition, necrosis, and edema. pH and metabolic imaging point out potential differences between subtypes investigated in this study. pH imaging using ^13^C‐labeled, deuterated zymonic acid as a probe was demonstrated for the first time in cats. While pH measurements in this study were not validated by gold standards such a blood gas analysis or any other imaging readouts such as ^31^P MRSI or CEST, zymonic acid as a pH sensor was carefully validated against other techniques in previous studies [22]. pH imaging in this work revealed pronounced acidification hotspots in tumors and overall tumor acidification for several subtypes. Nevertheless, mean pH values reported in this study contain weighting from non‐acidified vessel compartments, which explains why absolute pH values found in this study appear less acidified compared to previous studies of sarcoma pH [55, 56, 57]. Observed tumor acidification aligns with previous findings from canine sarcoma patients, where tumor acidification was observed to be an indicator of aggressiveness and poor prognosis [58]. While UPSs are generally considered highly aggressive [47], OS and CS have already been suggested to acidify their extracellular space via an increased expression of CAIX and vATPase [46]. This matches our observation of strong acidification and low metabolic flux in the OS case of this study. This observation supports the rationale for measuring pH directly rather than inferring it from metabolism alone.

AUCr values to quantify metabolism in this study also indicated strong metabolic flux from pyruvate to lactate in most sarcoma cases, albeit with marked heterogeneity across tumors and within lesions. AUCr‐based quantification provided a practical summary metric for the present acquisition given the low temporal resolution and the limited number of signal‐containing data points. However, variability might be reduced by kinetic modeling of dynamic imaging data [59] and acquisition at higher spatial and temporal resolution [60, 61]. Even with these limitations, the observed lactate production is consistent with the broader concept of metabolic rewiring in sarcomas [62]. One reason might be that despite an increased need for glucose and oxygen consumption, glucose oxidation was found to be impaired in sarcomas [63].

PET imaging of [^18^F]FMISO could be well integrated into the hyperpolarized ^13^C MRSI workflow, enabling additional hypoxia imaging within the imaging session. Arterial input function quantification suffered from the insertion of the dual‐tuned MRI coil during the PET acquisition, which induced further attenuation. However, neither kinetic modeling nor static averaging of late time points are notably affected by this technical burden, as coil insertion took place after the input function peak and prior to the tumor time activity curve section which was used for modeling. Hypoxia was indicated by elevated SUV and K_i_ values compared to healthy tissue and corroborated by pimonidazole staining, which matches previous studies of PET‐based hypoxia imaging in feline sarcoma patients [64, 65]. Unexpectedly, correlation of pimonidazole analysis and PET parameters remains limited, potentially due to different scales and slice thicknesses—consistent with known challenges in quantitatively linking PET hypoxia tracers to *ex vivo* hypoxia markers [66]. While SUV shows a slightly stronger correlation with pimonidazole compared to K_i_, the prognosis and therapy outcome have been shown to be indifferent between parameters [67]. Similar to our results, clinical studies enrolling sarcoma patients for [^18^F]FMISO PET point toward a relation between sarcoma aggressiveness and hypoxia, but evidence for this is still in its infancy [65, 68].

While PET hypoxia biomarkers were not conclusive as a predictor for patient outcome in this study, intratumoral heterogeneity is best captured by metabolic imaging using [1‐^13^C]pyruvate. Tumor acidification measured by hyperpolarized ^13^C MRSI of pH using [1,5‐^13^C_2_,3,6,6,6‐D_4_]zymonic acid turned out to be a promising predictive biomarker for patient outcome among all investigated parameters. This is consistent with the clinical motivation which assumes that extracellular acidity shapes antitumor immunity and may represent a modifiable vulnerability. If confirmed in larger studies, pH imaging could complement hypoxia and metabolic readouts for stratification and therapy monitoring, and could help identify tumors most likely to benefit from microenvironment‐targeted conditioning strategies.

One major limitation of this study is the low number of patients for each subtype, several of them including only one patient case. Further, as has been shown for the bimorphic tumor, subtype differentiation, even with comprehensive histopathology, is not always possible in veterinary patients. A main challenge here is the limited availability of feline species‐specific immunohistochemistry markers in contrast with human diagnostics. This restricts the ability to further characterize compartmental subtypes based on imaging. In addition, collecting all data with sufficient quality to perform multi‐parameter analysis is challenging and requires initial experience and optimization. This further limits the number of complete datasets.

Prior to this work, studies had already been conducted using combined hyperpolarized ^13^C MRSI and PET. These found correlations between lactate production and [^18^F]FDG [30] and further allowed to differentiate between bone sarcomas and soft tissue sarcomas in canine cancer patients [31]. Within these, also differences in correlation of the main parameters *k*_pl_ and FDG uptake were observed between subtypes. However, no general pattern across different tumors was found, confirming the high diversity and metabolic complexity of sarcomas. Similar results were also found in a study in rats [27], where cellularity and necrosis were considered to be important, potentially confounding factors. In addition, a combination of multiple modalities might be more reliable in better assessing early therapy response [69]. Beyond hyperpolarized [1‐^13^C]pyruvate and [^18^F]FDG, which are sometimes considered to be in competition [70], truly complementary imaging in a multimodal setting has already been performed to assess metabolism in conjunction with vascularization or receptor expression [32, 33]. Moreover, in particular PET/MRI has been shown to be of great value in understanding complex diseases and exploiting the versatility of multimodal imaging by combining PET and functional MRI in Alzheimer's disease [71, 72, 73]. The advances in imaging hardware development over the last decades led to the emergence of a variety of powerful multimodal devices. These range across scales from in vitro platforms working on a single‐cell level [74] to clinical routine in humans [75]. PET and molecular imaging by MRI are sometimes considered to be opponents in the dominance for disease diagnosis and therapy decision making. Instead, it becomes clear that a multimodal approach exploiting their complementarity provides additional value for diagnosis and therapy response assessment.

## 4 Conclusion

In summary, this work advances translational multimodal imaging by implementing a single‐session, clinically realistic protocol on a 3 T PET/MRI system that simultaneously measures hypoxia ([^18^F]FMISO), metabolic flux (hyperpolarized [1‐^13^C]pyruvate), and extracellular pH (hyperpolarized [1,5‐^13^C_2_,3,6,6,6‐D_4_]zymonic acid). Feline sarcomas offer a pragmatic bridge between rodent studies and human application by enabling evaluation of feasibility, co‐localization, and heterogeneity in a clinical workflow setting. Notably, pH mapping proved to be of value for characterizing intra‐ and intertumoral heterogeneity and predicting patient outcome. If validated in larger cohorts, this approach could support biomarker development for stratification and therapy monitoring.

## 5 Experimental Section

### 5.1 Study Design and Workflow

In this study, the feasibility and tolerability of simultaneous hyperpolarized ^13^C MRSI and PET were assessed to study hypoxia, metabolism, and tumor acidification in direct comparison. The experiments were performed according to pertinent laws and regulations and approved by an ethical review board (Regierung von Oberbayern, ROB‐55.2‐2532.Vet_02‐17‐177). Patients were recruited via a newsletter that was sent to a large number of veterinary practices in Bavaria, Germany. Pet cats whose owners expressed interest in the study and provided written informed consent, and which had a palpable mass underwent a biopsy, a blood count, a cardiopulmonary check, and an ultrasound exam. Inclusion criteria were a confirmed, curatively resectable malignant mesenchymal mass of at least 2 cm in palpable diameter. Recurrent or metastatic tumors were exclusion criteria. Prior to the multimodal imaging protocol, written informed consent on risks and conditions of the study was collected from the owners. The age, sex, breed, and weight of all included patients are listed in Table S1. Blood counting was performed directly before and after imaging to assess immediate adverse side effects. Animals were weighed, handled, and monitored continuously by at least one experienced veterinarian at all times during the imaging session. For the injection of imaging agents, a catheter was placed in one of the forelimbs. Anesthesia was initiated by intravenous injection of propofol. Cats were intubated, and anesthesia was maintained by controlled ventilation using isoflurane (0.8%–2.8%) with oxygen‐enriched (40%) medical air as carrier gas. Body temperature was maintained by blowing warm air into the bore (Mistral air, The 37C Company, Amersfoort, Netherlands). Rectal temperature was monitored using an MR‐compatible temperature monitoring system Model 1030 (SA Instruments, Stony Brook, USA). Heart rate and peripheral blood oxygenation (SpO_2_) were monitored using a MR‐compatible patient monitor (Expression MR Patient Monitor, Invivo, Gainesville, USA). Additionally, end‐tidal CO_2_ (EtCO_2_), inspiratory peak pressure, and respiratory minute volume were continuously monitored. Following imaging and patient wake‐up, cats were returned to their owners in accordance with radiation protection regulations. For surgery planning, anatomical MR images were diagnosed by experienced clinical human radiologists and veterinary radiologists. All cats underwent surgery for tumor removal, generally 3–5 days post imaging and received no further tumor‐related treatment within the follow‐up period. Resected tumor specimens were histopathologically evaluated to confirm complete tumor removal (R0 status). After histopathological workup, all resected tumors were microscopically evaluated by experienced pathologists. A complete list of tumor types, locations, extensions, and volumes is provided in Table S1.

### 5.2 Hyperpolarization

For pH imaging, 213.4 mg [1,5‐^13^C_2_,3,6,6,6‐D_4_]zymonic acid (synthesized in‐house) [44] was mixed with 8.5 mg OX063 trityl radical (GE Healthcare, Chicago, USA) and added to 190 µl dimethyl sulfoxide. The mixture was vortexed for 90 min, transferred to a DNP sample cup, and frozen in liquid nitrogen. For co‐polarization with [^13^C]urea, 135 µL of a 10 m [^13^C]urea in glycerol containing 30 mm OX063 was added, and the sample cup was again frozen in liquid nitrogen. The sample was then polarized by DNP in a HyperSense DNP polarizer (Oxford Instruments, Abingdon, UK) at a microwave frequency of 94.190 GHz and 100 mW power for at least 3–4 h. Having reached saturation polarization, dissolution was performed using 9 mL dissolution agent being heated to 180°C. It consisted of 7.65 mL D_2_O containing 80 mm tris(hydroxymethyl)aminomethane (TRIS), 0.1 g/L Na_2_‐ethylenediaminetetraacetic acid (EDTA), to which 1.35 mL 1 m NaOH in D_2_O was added. The resulting injection solutions contained concentrations of 150 mm zymonic acid and 150 mm urea with pH 8.08 ± 1.78. For imaging metabolism, 96 µL of a solution containing 14 m [1‐^13^C]pyruvic acid (Sigma–Aldrich, St. Louis, USA), 15 mm OX063 and 1 mm DOTAREM (Guerbet, Villepinte, France) was added to a DNP sample cup and polarized by DNP in a HyperSense DNP polarizer at a microwave frequency of 94.190 GHz and 100 mW power for at least 30 min. The sample was dissolved using a mixture of 8.307 mL H_2_O buffered with 80 mm TRIS and 0.1 g/L Na_2_EDTA and 0.693 mL 1 m NaOH in H_2_O. The resulting injection solutions contained 150 mm pyruvic acid with pH 6.06 ± 1.73.

### 5.3 Imaging Protocol

Imaging was performed on a clinical 3 T PET/MRI scanner (Biograph mMR, Siemens Healthcare, Erlangen, Germany) using syngo VE11P service pack 3. Following the initiation of anesthesia, cats were centered with the tumor pointing downward on an in‐house‐built bed mounted on the patient bed of the clinical scanner. Initial localizer scans, attenuation maps, and whole‐body 3D imaging (Experimental Section ‘Anatomical MRI’) were acquired using the scanner's body coil. For subsequent ^13^C MRSI (Experimental Section ‘^13^C MRSI’), a dual‐tuned flexible ^1^H/^13^C Tx/Rx surface coil with 21 cm × 32 cm cross section (Rapid Biomedical, Rimpar, Germany) was inserted on the cat bed rail system to place the coil directly under the tumor without moving the cat.

### 5.4 Anatomical MRI

Initial patient positioning was verified using a standard localizer sequence. Parallel to start of the PET acquisition, a standard generalized autocalibrating partially parallel acquisitions (GRAPPA)‐based 3D fast low‐angle shot (FLASH) two‐echo Dixon sequence [76, 77, 78] with matrix 126 × 192 × 128, resolution 2.64 × 2.64 × 3.12 mm^3^, TR 3.56 ms, TE 1.23 ms, total acquisition time 31.34 s was used for fat‐water‐based attenuation mapping. Whole‐body 3D imaging for primary tumor localization and detection of potential additional lesions used a 3D magnetization‐prepared rapid gradient echo (MPRAGE) sequence with typical parameters being matrix 120 × 320 × 192, resolution 0.5 × 0.5 × 1 mm^3^, TR 2050 ms, inversion time (TI) 1040 ms, TE 4.16 ms, 4 averages, total acquisition time 8 min 11 s. A second 3D sequence, namely, a 3D fluid‐attenuated inversion recovery (FLAIR) was acquired with typical parameters being matrix 144 × 384 × 192, resolution 0.83 × 0.83 × 1 mm^3^, TR 3000 ms, TI 1800 ms, TE 393 ms, echo train length 258, 4 averages, total acquisition time 8 min 21 s. Additionally, for MRSI slice positioning, axial, sagittal, and coronal 2D FLASH *T*_1_‐weighted images with typical parameters being matrix 216 × 384 × 35, slice thickness 3 mm, resolution 0.41 × 0.41 × 3 mm^3^, TR 250 ms, TE 2.84 ms, 12 averages, total acquisition time 4 min 47 s were acquired. For anatomical coregistration for MRSI slices, *T*_2_‐weighted images using half‐Fourier acquisition single‐shot turbo spin echo imaging (HASTE) with matrix 144 × 384 × 3, slice thickness 3 mm, resolution 0.41 × 0.41 × 3 mm^3^, slice spacing 4.98 mm, TR 2000 ms, TE 49 ms, 32 averages, total acquisition time 3 min 6 s were acquired.

### 5.5 ^13^C MRSI

Shimming was performed using the routine user‐interactive map shim tool and the ^13^C frequency was calibrated using an 8 M [^13^C]urea phantom doped with Gd‐DOTA. The coil reference voltage was manually adjusted by searching for the reference voltage returning the highest ^13^C signal in single ^13^C spectra of the urea phantom. For hyperpolarized imaging of pH, cats were injected with 8 mL of hyperpolarized zymonic acid‐urea solution, with injections starting 38 ± 3 s post dissolution and injection duration 14 ± 2 s. Free induction decay chemical shift imaging (FIDCSI) for pH imaging was started with the end of injection using a 15° flip angle, matrix 8 × 16 with elliptical k‐space encoding, FOV 80 × 160 mm^2^, slice thickness 13 mm, in‐plane resolution 10 × 10 mm^2^, TR 156 ms, spectral receive bandwidth 4000 Hz, 256 spectral points, acquisition time 10.14 s as typical parameters. For hyperpolarized imaging of pyruvate‐lactate label exchange, 8 mL of hyperpolarized pyruvate solution were injected 38 ± 6 s post dissolution and injection duration 14 ± 3 s at a delay 55 ± 11 min post zymonic acid injection. Dynamic CSI used the same parameters as for hyperpolarized imaging of pH, except being acquired for a total of 2 × 8 frames, leading to 16 frames overall with a typical temporal resolution of 10.14 s and total acquisition time of 162.24 s. Acquisition started with the start of injection.

### 5.6 Diffusion‐Weighted Imaging

DWI used a standard 2D echo‐planar imaging (EPI) sequence ’ep2d’ with typical parameters being: matrix 60 × 128 × 3, slice thickness 3 mm, resolution 1.5 × 1.5 × 3 mm^3^, slice spacing 4.98 mm, TR 3000 ms, TE 49 ms, 4 averages, total acquisition time 7 min 36 s. For diffusion encoding, 8 *b*‐value (0, 50, 100, 200, 300, 500, 800, 1000 s/mm^2^) images were acquired, the latter two being averaged 8‐fold. *b*‐value images were fitted voxelwise using a mono‐exponential with offset to derive ADC values. Voxels with less than the two‐fold median signal intensity and non‐converging ADC fits in the range 0–5·10^−3^ mm^2^/s were discarded.

### 5.7 PET Imaging

For PET imaging, PET data were acquired as a large list mode file for a total of 180 min. Due to file size constraints, the PET acquisition was split into a first block lasting 60 min and a second block lasting 120 min. Acquisition started right before injection of 149.7 ± 7.6 MBq [^18^F]FMISO dissolved in 2 mL citrate‐free solution (ABX, Radeberg, Germany). PET images from both blocks were reconstructed separately using the GRAPPA attenuation map and framing: 20 × 3 s, 12 × 5 s, 26 × 30 s, 3 × 300 s, 3 × 600 s for the first block and framing: 12 × 600 s for the second block. Digital imaging and communications in medicine (DICOM) images from both blocks were merged into one joint series using an in‐house written Python script.

### 5.8 Histopathology

One hour prior to surgery, cats were injected with 0.3 g/m^2^ pimonidazole (Hypoxyprobe Inc., Burlington, USA) for immunohistochemical visualization of hypoxia. Following surgery, tumors were marked with tissue ink of various colors to encode absolute orientation and properly fixed in formalin (1:10 tissue:formalin ratio). For coregistration with imaging data, tumors were cut into 0.5 cm thick slices according to the ^13^C MRSI slice orientation and smaller patches for each slice to fit histology cassettes (41 mm × 27.5 mm × 12 mm, Carl Roth, #EE16.1). Tumors were then stained with H&E according to a standard protocol. Fluorescein isothiocyanate (FITC)‐labeled anti‐pimonidazole antibody (Hypoxyprobe Inc., #HP2‐1000Kit, primary antibody: Lot# 05.18.18, secondary antibody: Lot# 08.23.18) followed by a horseradish peroxidase (HRP)‐conjugated anti‐FITC secondary antibody was used in order to immunohistochemically visualize and confirm tissue hypoxia detected by [^18^F]FMISO PET. Submitted routine diagnostic samples of feline FSs from cat patients not undergoing pimonidaziol injection were used as negative controls (Figure S3). Based on H&E histomorphology and, if applicable, immunohistochemical marker profile, tumors were then classified according to the current veterinary WHO classifications and veterinary pathology standards [45, 79, 80]. Furthermore, FSs were graded (G1 = low grade, G2 = intermediate, G3 = high grade) according to a grading guideline for feline FSs [40].

### 5.9 Blood Counting

For each patient, at least two blood samples (one prior and one post imaging) and up to four blood samples were drawn into S‐Monovette EDTA K3E (Sarstedt, Sarstedt, Germany). Blood counting was performed using a Sysmex XT‐2000iVet (Sysmex, Kobe, Japan). Blood serum was analyzed using a Roche Cobas Integra 400 plus (Roche, Basel, Switzerland).

### 5.10 Follow‐Up Survey

To assess patient outcome following surgical tumor removal, a follow‐up survey was conducted to interrogate for subsequent treatments related to the diagnosed tumor, recurrence, and survival status. For this purpose, cat patient owners were contacted via phone between July 2024 and September 2024. The survey form is displayed in Figure S5. The survey was completed by nine cat patient owners, and overall survival for one patient was obtained from a follow‐up visit at the Centre of Clinical Veterinary Medicine. For survival analysis, patients with follow‐up information were dichotomized into favorable and unfavorable prognosis groups. To determine the optimal parameter‐specific cutoff value, ROC curve analysis was performed for three different time points (6, 12, 18 months), and optimal cutoffs were selected for each ROC curve by calculating the Youden index. Derived cutoff values were then averaged across all three time points to yield the final cutoff value. Significance, determined by *p* values ≤ 0.05, was tested using the Mantel‐Cox test.

### 5.11 Data Analysis

Generation of scripts in MATLAB (The MathWorks, Natick, USA) was assisted by a large language model (ChatGPT, OpenAI, San Francisco, USA). No blinding was applied at any stage because of the explorative nature of this pilot study. For coregistration of all imaging modalities, DWI and PET images and related parametric maps were downsampled to match the resolution of the four‐fold interpolated ^13^C acquisitions.

### 5.12 pH Map Reconstruction

For the reconstruction of pH maps, raw data files were exported via TWIX. All post‐processing was performed in MATLAB 2022b using a graphical user interface (GUI) platform similar to the one described previously [23]. In detail, CSI raw data was zero‐filled by a factor of 2 in spectral dimension, interpolated by a factor 4 in spatial directions, and line broadened by 6 Hz. For pH fitting, [^13^C]urea and up to four zymonic acid peaks (C_1_ and C_5_) were searched for. The latter correspond to two compartments, with compartment ranges being 7.25–7.9 and 6.6–7.25 based on previous findings [22, 23], with a minimum signal‐to‐noise ratio (SNR) of 20 relative to apodized spectra edges. pH values were fitted from peak positions relative to urea using the reported calibration curve [22]. For voxels containing more than one pH compartment, mean pH values, only reported as pH throughout this study, were averaged using the peak intensities as weights. Tumor mean pH values were averaged across the whole‐tumor ROI. Acidification ΔpH was defined and calculated by subtracting the tumor pH (pH_tumor_) from the pH of an adjacent muscle region (pH_muscle_) within the same subject and serves only for intertumor comparison:

$$\Delta pH=pH_{muscle\;}-pH_{tumor}$$

### 5.13 Quantification of Pyruvate‐Lactate Label Exchange

For quantification of pyruvate‐lactate label exchange, raw data files were exported via TWIX. All post‐processing was performed in MATLAB 2022b using a GUI platform similar to the one described previously [23]. In detail, CSI raw data was zero‐filled by a factor of 2 in spectral dimension, interpolated by a factor 4 in spatial directions, and line broadened by 6 Hz. [1‐^13^C]Pyruvate and [1‐^13^C]lactate peak positions were integrated over a range of 16 Hz, and the resulting time curves were baseline‐corrected. Pyruvate and lactate time curves were then summed over time, and the cumulative lactate signal was divided by the cumulative pyruvate signal to obtain AUCr values. Voxels with a pyruvate peak SNR of less than 5 were excluded.

### 5.14 Quantification of Hypoxia

Quantification and post‐processing of PET data were done using Inveon Research Workplace 4.2 (Siemens Healthineers, Erlangen, Germany). PET images, anatomical 3D and 2D MRSI‐volume matching MR images were imported, and coregistration manually fine‐adjusted if necessary using rigid rotation or translation. PET signal intensities were converted to SUVs, and intensity values from the last 30 min, corresponding to three frames, were averaged. This derives a static late uptake value as a measure for hypoxia as commonly used in clinical studies [81]. As a second measure to quantify hypoxia, late uptake phases, which show linear uptake kinetics, were modeled using the Patlak model [82]. For pharmacokinetic modeling, regions of interest were drawn on a vena cava segment close to the tumor location, and the extracted time curves served as image‐derived input functions for modeling. Using this input function and the dynamic curves from t = 60 min to t = 180 min (second acquisition block) for voxelwise kinetic modeling yielded kinetic uptake rate (K_i_) maps, where positive values indicate an increasing degree of hypoxia [66].

### 5.15 Quantification of Immunohistochemical Staining

Scanned slides were analyzed in QuPath 0.5.1 [83]. First color deconvolution was achieved by adjusting staining vectors for diaminobenzidine and haematoxylin separately for each slide individually using strongly positive and negative section regions. Calculation of the hypoxia‐positive cell fraction was performed using the built‐in positive cell detection algorithm with a binary threshold. The binary threshold was determined by control classification on positive and negative control sections. Detection and classification parameters for each immunohistochemical staining are listed in Figure S4G.

### 5.16 Statistics and Principal Component Analysis

Statistical analysis was performed using GraphPad Prism 7 (GraphPad Software, San Diego, USA), and two‐sided *t*‐tests or Mann–Whitney U‐tests were used depending on whether datasets were normally distributed. Two‐parameter linear regression analysis was performed in Origin 2018b (OriginLab, Northampton, USA). Values of *p* ≤ 0.05 were considered statistically significant. For intertumor correlation matrices, whole‐tumor mean parameter values or individual voxel parameters were plotted pairwise and fitted using linear regression. For intratumoral correlation analysis, ADC values were summed voxelwise across all three imaged slices, and PET and DWI parametric maps were resampled to match the resolution of the interpolated ^13^C parameter maps. For multidimensional analysis of parameter relationships, PCA was performed using the built‐in MATLAB algorithm on either three (pH/Ac., AUCr, SUV) or four (pH/Ac., AUCr, SUV, ADC) parameters for subjects with a complete set of valid imaging data. For qualitative visualization of general parameter dependencies, non‐standardized parameters were used, and the first eigenvector axis was displayed (as shown in Figure 7C). For quantitative analysis (Figure 7D–H), z‐scores [84, 85] were calculated for each parameter using a global median and interquartile range across all tumors. To assess the degree of hypoxia‐driven lactate metabolism and the associated extracellular acidosis within a tumor, a ‘pH‐metabolism‐hypoxia (PMH) score’ was defined as the absolute value of the dot product between the first PCA vector and a normalized ‘Warburg vector’ *W* = [‐1, 1, 1]/$\sqrt{3}$.

### 5.17 Subset‐Based PCA Analysis of Intratumoral Heterogeneity

To identify the two or single most informative imaging modality for capturing tumor heterogeneity, a projection‐based subset evaluation approach inspired by the principal variables concept was used [86, 87, 88]. To determine the single most descriptive modality, for each tumor, each parameter (pH, AUCr, K_i_, ADC) was evaluated separately with respect to its ability to preserve the dominant multivariate heterogeneity pattern defined by PCA of the full parameter set within a tumor. For this purpose, 5‐fold cross‐validation was performed by partitioning the voxel data of each tumor into five approximately equal subsets. In each iteration, four of these fractions *S_fit_* were used to perform a linear least‐squares regression with offset of the included PCA scores S_fit_ and the z‐scores of the tested modality, and the remaining subset *S_test_* was held out for testing.

The fitted regression coefficients were then applied to the held‐out test subset to predict the corresponding PCA scores ${\hat{S}}_{test}$. The predictive accuracy was quantified using the coefficient of determination:

$$R^{2}=1-\frac{SSE}{SST}$$

where $SSE=\sum {\left(S_{test}-{\hat{S}}_{test}\right)}^{2}$ is the residual sum of squares between true and predicted PCA scores in the test subset, and $SST=\sum {\left(S_{test}-{\bar{S}}_{fit}\right)}^{2}$ is the total sum of squares relative to the mean PCA score of the subsets for fitting. This procedure was repeated five times so that each subset served once as test data, and the resulting *R* ^2^ values were averaged. The modality with the highest mean *R* ^2^ was considered to best preserve the dominant PCA‐defined heterogeneity structure of the tumor. To determine the two most descriptive modalities, the same workflow as for one modality was applied but employing the z‐scores from two modalities for linear regression. The analysis was repeated for all pairs of parameters, and the best pair was determined from the largest value of *R* ^2^ across all parameter pairs within a tumor.

## Author Contributions

Conceptualization: MS, KS, AML, JH, CB, FS. Methodology: MG, TG, SB, JC, MS, KS, AML, JH, FS. Investigation: MG, TG, SK, SB, EB, FHAvH, PW. Formal analysis: MG, TG, AW, JC, SN, CL, KS, FS. Visualization: MG, TG, FS. Funding acquisition: MS, WW, KS, AML, JH, CB, FS. Project administration: WW, KS, AML, JH, CB, FS. Supervision: JH, FS. Writing – original draft: MG, TG, FS. Writing – review and editing: MG, TG, AW, PW, FS.

## Funding

This research was funded by Deutsche Forschungsgemeinschaft (DFG, German Research Foundation, Sonderforschungsbereich (SFB) 824, subprojects A7, Z2 and Z3, the Emmy Noether programme grant number 391523415 (MS, WW, KS, FS). Young Academy of the Bavarian Academy of Sciences and Humanities (FS). European Union's Horizon 2020 research and innovation program under grant agreement No 820374 (FS). German Federal Ministry of Research, Technology and Space (BMFTR) in the Funding Program ’Quantum Technologies – from Basic Research to Market’ Under the Project ’QuE‐MRT’, contract number: 13N16450 (FS).

## Conflicts of Interest

FS serves on the scientific advisory board of NVision Imaging Technologies GmbH.

## Supporting information




**Supporting File**: advs77830‐sup‐0001‐SuppMat.docx.

## Data Availability

The data that support the findings of this study are available from the corresponding author upon reasonable request.

## References

[1] D. Hanahan and R. A. Weinberg , “Hallmarks of Cancer: The Next Generation,” Cell 144, no. 5 (2011): 646–674, 10.1016/j.cell.2011.02.013. 21376230

[2] O. Warburg , “The Metabolism of Carcinoma Cells,” The Journal of Cancer Research 9 (1925): 148–163, 10.1158/jcr.1925.148.

[3] G. L. Semenza , “HIF‐1: Upstream and Downstream of Cancer Metabolism,” Current Opinion in Genetics & Development 20, no. 1 (2010): 51–56, 10.1016/j.gde.2009.10.009. PMC2822127 19942427

[4] J. R. Griffiths , “Are Cancer Cells Acidic?,” British Journal of Cancer 64 (1991): 425–427, 10.1038/bjc.1991.326. PMC1977628 1911181

[5] V. Huber , C. Camisaschi , A. Berzi , et al., “Cancer Acidity: An Ultimate Frontier of Tumor Immune Escape and a Novel Target of Immunomodulation,” Seminars in Cancer Biology 43 (2017): 74–89, 10.1016/j.semcancer.2017.03.001. 28267587

[6] S. Pilon‐Thomas , K. N. Kodumudi , A. E. El‐Kenawi , et al., “Neutralization of Tumor Acidity Improves Antitumor Responses to Immunotherapy,” Cancer Research 76 (2016): 1381–1390, 10.1158/0008-5472.CAN-15-1743. PMC4829106 26719539

[7] H. Wu , V. Estrella , M. Beatty , et al., “T‐Cells Produce Acidic Niches in Lymph Nodes to Suppress Their Own Effector Functions,” Nature Communications 11, no. 1 (2020): 4113, 10.1038/s41467-020-17756-7. PMC7431837 32807791

[8] S. H. Lee and J. R. Griffiths , “How and Why Are Cancers Acidic? Carbonic Anhydrase IX and the Homeostatic Control of Tumour Extracellular pH,” Cancers 12 (2020): 1616, 10.3390/cancers12061616. PMC7352839 32570870

[9] P. Irrera , L. Consolino , M. Roberto , et al., “In Vivo MRI‐CEST Tumor pH Imaging Detects Resistance to Proton Pump Inhibitors in Human Prostate Cancer Murine Models,” Cancers 14, no. 19 (2022): 4916, 10.3390/cancers14194916. PMC9563279 36230838

[10] R. A. Gatenby and R. J. Gillies , “Why do Cancers Have High Aerobic Glycolysis?,” Nature Reviews Cancer 4, no. 11 (2004): 891–899, 10.1038/nrc1478. 15516961

[11] R. J. Harris , J. Yao , A. Chakhoyan , et al., “Simultaneous pH ‐Sensitive and Oxygen‐Sensitive MRI of Human Gliomas at 3 T Using Multi‐Echo Amine Proton Chemical Exchange Saturation Transfer Spin‐and‐Gradient Echo Echo‐Planar Imaging ( CEST‐SAGE‐EPI ),” Magnetic Resonance in Medicine 80, no. 5 (2018): 1962–1978, 10.1002/mrm.27204. PMC6107417 29626359

[12] J. Yao , A. Hagiwara , T. C. Oughourlian , et al., “Diagnostic and Prognostic Value of pH‐ and Oxygen‐Sensitive Magnetic Resonance Imaging in Glioma: A Retrospective Study,” Cancers 14, no. 10 (2022): 2520, 10.3390/cancers14102520. PMC9139712 35626127

[13] J. M. Goldenberg , J. Cárdenas‐Rodríguez , and M. D. Pagel , “Preliminary Results that Assess Metformin Treatment in a Preclinical Model of Pancreatic Cancer Using Simultaneous [^18^F]FDG PET and acidoCEST MRI,” Molecular Imaging and Biology 20, no. 4 (2018): 575–583, 10.1007/s11307-018-1164-4. PMC6043393 29374343

[14] D. L. Longo , A. Bartoli , L. Consolino , et al., “In Vivo Imaging of Tumor Metabolism and Acidosis by Combining PET and MRI‐CEST pH Imaging,” Cancer Research 76, no. 22 (2016): 6463–6470, 10.1158/0008-5472.CAN-16-0825. 27651313

[15] Q. Qi , M. S. Fox , H. Lim , et al., “Glucose Infusion Induced Change in Intracellular pH and Its Relationship With Tumor Glycolysis in a C6 Rat Model of Glioblastoma,” Molecular Imaging and Biology 25, no. 2 (2023): 271–282, 10.1007/s11307-022-01726-0. 36418769

[16] M. A. Neveu , G. De Preter , V. Marchand , et al., “Multimodality Imaging Identifies Distinct Metabolic Profiles In Vitro and In Vivo,” Neoplasia 18, no. 12 (2016): 742–752, 10.1016/j.neo.2016.10.010. PMC5126136 27889643

[17] A. Hagiwara , J. Yao , C. Raymond , et al., ““Aerobic Glycolytic Imaging” of Human Gliomas Using Combined pH‐, Oxygen‐, and Perfusion‐Weighted Magnetic Resonance Imaging,” NeuroImage: Clinical 32 (2021): 102882, 10.1016/j.nicl.2021.102882. PMC8609049 34911188

[18] P. Andrzejewski , G. Wengert , T. H. Helbich , et al., “Sequential [^18^F]FDG‐[^18^F]FMISO PET and Multiparametric MRI at 3T for Insights Into Breast Cancer Heterogeneity and Correlation With Patient Outcomes: First Clinical Experience,” Contrast Media & Molecular Imaging 2019 (2019): 1–9, 10.1155/2019/1307247. PMC6341235 30728757

[19] W. J. Koh , J. S. Rasey , M. L. Evans , et al., “Imaging of Hypoxia in Human Tumors With [F‐18]fluoromisonidazole,” International Journal of Radiation Oncology*Biology*Physics 22, no. 1 (1992): 199–212, 10.1016/0360-3016(92)91001-4. 1727119

[20] S. J. Nelson , J. Kurhanewicz , D. B. Vigneron , et al., “Metabolic imaging of Patients With Prostate Cancer using Hyperpolarized [1‐^13^C]Pyruvate,” Science Translational Medicine 5 (2013): 198ra108, 10.1126/scitranslmed.3006070. PMC4201045 23946197

[21] K. Golman , R. in't Zandt , and M. Thaning , “Real‐Time Metabolic Imaging,” Proceedings of the National Academy of Sciences 103, no. 30 (2006): 11270–11275, 10.1073/pnas.0601319103. PMC1544077 16837573

[22] S. Düwel , C. Hundshammer , M. Gersch , et al., “Imaging of pH In Vivo Using Hyperpolarized ^13^C‐Labelled Zymonic Acid,” Nature Communications 8 (2017): 15126, 10.1038/ncomms15126. PMC5482723 28492229

[23] M. Grashei , P. Wodtke , J. G. Skinner , et al., “Simultaneous Magnetic Resonance Imaging of pH, Perfusion and Renal Filtration Using Hyperpolarized ^13^C‐Labelled Z‐OMPD,” Nature Communications 14, no. 1 (2023): 5060, 10.1038/s41467-023-40747-3. PMC10442412 37604826

[24] F. A. Gallagher , M. I. Kettunen , S. E. Day , et al., “Magnetic Resonance Imaging of pH In Vivo Using Hyperpolarized ^13^C‐Labelled Bicarbonate,” Nature 453, no. 7197 (2008): 940–943, 10.1038/nature07017. 18509335

[25] C. Hundshammer , S. Düwel , and F. Schilling , “Imaging of Extracellular pH Using Hyperpolarized Molecules,” Israel Journal of Chemistry 57 (2017): 788–799, 10.1002/ijch.201700017.

[26] A. Eldirdiri , A. Clemmensen , S. Bowen , A. Kjær , and J. H. Ardenkjær‐Larsen , “Simultaneous Imaging of Hyperpolarized [1,4‐^13^C_2_]fumarate, [1‐^13^C]pyruvate and ^18^F–FDG in a Rat Model of Necrosis in a Clinical PET/MR Scanner,” NMR in Biomedicine 30, no. 12 (2017): 3803, 10.1002/nbm.3803. 29044751

[27] C. Hundshammer , M. Braeuer , C. A. Müller , et al., “Simultaneous Characterization of Tumor Cellularity and the Warburg Effect With PET, MRI and Hyperpolarized ^13^C‐MRSI,” Theranostics 8 (2018): 4765–4780, 10.7150/thno.25162. PMC6160766 30279736

[28] M. I. Menzel , E. V. Farrell , M. A. Janich , et al., “Multimodal Assessment of In Vivo Metabolism With Hyperpolarized [1‐^13^C]MR Spectroscopy and ^18^F‐FDG PET Imaging in Hepatocellular Carcinoma Tumor–Bearing Rats,” Journal of Nuclear Medicine 54, no. 7 (2013): 1113–1119, 10.2967/jnumed.112.110825. 23596002

[29] H. Gutte , A. E. Hansen , S. T. Henriksen , et al., “Simultaneous Hyperpolarized ^13^C‐pyruvate MRI and ^18^F‐FDG‐PET in Cancer (hyperPET): Feasibility of a New Imaging Concept Using a Clinical PET/MRI Scanner,” American Journal of Nuclear Medicine and Molecular Imaging 5, no. 1 (2015): 38–45. PMC4299777 25625025

[30] H. Gutte , A. E. Hansen , M. M. E. Larsen , et al., “Simultaneous Hyperpolarized ^13^C‐Pyruvate MRI and^18^F‐FDG PET (HyperPET) in 10 Dogs With Cancer,” Journal of Nuclear Medicine 56, no. 11 (2015): 1786–1792, 10.2967/jnumed.115.156364. 26338899

[31] A. E. Hansen , H. Gutte , P. Holst , et al., “Combined Hyperpolarized ^13^C‐pyruvate MRS and ^18^F‐FDG PET (hyperPET) Estimates of Glycolysis in Canine Cancer Patients,” European Journal of Radiology 103 (2018): 6–12, 10.1016/j.ejrad.2018.02.028. 29803387

[32] A. Clemmensen , A. E. Hansen , P. Holst , et al., “[^68^Ga]Ga‐NODAGA‐E[(cRGDyK)]_2_ PET and hyperpolarized [1‐^13^C] pyruvate MRSI (hyperPET) in Canine Cancer Patients: Simultaneous Imaging of Angiogenesis and the Warburg Effect,” European Journal of Nuclear Medicine and Molecular Imaging 48, no. 2 (2021): 395–405, 10.1007/s00259-020-04881-0. PMC7835292 32621132

[33] S. V. Bachawal , J. M. Park , K. S. Valluru , M. D. Loft , and S. A. Felt , “Multimodality Hyperpolarized C‐13 MRS/PET/Multiparametric MR Imaging for Detection and Image‐Guided Biopsy of Prostate Cancer: First Experience in a Canine Prostate Cancer Model,” Molecular Imaging and Biology 21, no. 5 (2019): 861–870, 10.1007/s11307-018-1235-6. 30793241

[34] G. Sharma , Hyperpolarized 13C Pyruvate‐MRI and FDG‐PET in a SingleExam for the Prognosis of Ischemic Cardiomyopathy (FDG PET‐HP MRI) (National Library of Medicine (NLM), 2025), https://clinicaltrials.gov/study/NCT06814587.

[35] S. Aime , D. D. Castelli , and E. Terreno , “Novel pH‐Reporter MRI Contrast Agents,” Angewandte Chemie (2002): 4334–4336, 10.1002/1521-3773(20021115)41:22<4334::AID-ANIE4334>3.0.CO;2-1. 12434381

[36] R. J. Gillies , N. Raghunand , M. L. Garcia‐Martin , and R. A. Gatenby , “pH Imaging,” IEEE Engineering in Medicine and Biology Magazine 23, no. 5 (2004): 57–64, 10.1109/MEMB.2004.1360409. 15565800

[37] A. I. Hashim , X. Zhang , J. W. Wojtkowiak , G. V. Martinez , and R. J. Gillies , “Imaging pH and Metastasis,” NMR in Biomedicine 24 (2011): 582–591, 10.1002/nbm.1644. PMC3740268 21387439

[38] C. R. White , “Allometric Estimation of Metabolic Rates in Animals,” Comparative Biochemistry and Physiology Part A: Molecular & Integrative Physiology 158, no. 3 (2011): 346–357, 10.1016/j.cbpa.2010.10.004. 20937406

[39] C. R. White and R. S. Seymour , “Mammalian Basal Metabolic Rate is Proportional to Body Mass^2/3^ ,” Proceedings of the National Academy of Sciences USA 100, no. 7 (2003): 4046–4049, 10.1073/pnas.0436428100. PMC153045 12637681

[40] S. S. Couto , S. M. Griffey , P. C. Duarte , and B. R. Madewell , “Feline Vaccine‐Associated Fibrosarcoma: Morphologic Distinctions,” Veterinary Pathology 39, no. 1 (2002): 33–41, 10.1354/vp.39-1-33. 12102217

[41] R. Graf , K. Grüntzig , M. Hässig , et al., “Swiss Feline Cancer Registry: A Retrospective Study of the Occurrence of Tumours in Cats in Switzerland From 1965 to 2008,” Journal of Comparative Pathology 153, no. 4 (2015): 266–277, 10.1016/j.jcpa.2015.08.007. 26422414

[42] M. Dobromylskyj , “Feline Soft Tissue Sarcomas: A Review of the Classification and Histological Grading, With Comparison to Human and Canine,” Animals 12, no. 20 (2022): 2736, 10.3390/ani12202736. PMC9597747 36290122

[43] G. L. Semenza , “HIF‐1 Mediates Metabolic Responses to Intratumoral Hypoxia and Oncogenic Mutations,” Journal of Clinical Investigation 123, no. 9 (2013): 3664–3671, 10.1172/JCI67230. PMC3754249 23999440

[44] C. Hundshammer , S. Düwel , S. S. Köcher , et al., “Deuteration of Hyperpolarized ^13^C‐Labeled Zymonic Acid Enables Sensitivity‐Enhanced Dynamic MRI of pH,” Chemphyschem 18 (2017): 2422–2425, 10.1002/cphc.201700779. 28719100

[45] P. Roccabianca , F. Y. Schulman , and G. Avallone , Surgical Pathology of Tumors of Domestic Animals Volume 3: Tumors of soft tissue (Davis‐Thompson DVM Foundation, 2020).

[46] G. Di Pompo , M. Cortini , N. Baldini , and S. Avnet , “Acid Microenvironment in Bone Sarcomas,” Cancers 13, no. 15 (2021): 3848, 10.3390/cancers13153848. PMC8345667 34359749

[47] M. Lehnhardt , A. Daigeler , H. H. Homann , et al., “MFH Revisited: Outcome After Surgical Treatment of Undifferentiated Pleomorphic or Not Otherwise Specified (NOS) Sarcomas of the Extremities—An Analysis of 140 Patients,” Langenbeck's Archives of Surgery 394, no. 2 (2009): 313–320, 10.1007/s00423-008-0368-5. 18584203

[48] P. Gouel , F. Callonnec , F. R. Obongo‐Anga , et al., “Quantitative MRI to Characterize Hypoxic Tumors in Comparison to FMISO PET/CT for Radiotherapy in Oropharynx Cancers,” Cancers 15, no. 6 (2023): 1918, 10.3390/cancers15061918. PMC10047588 36980806

[49] A. K. LeBlanc and C. N. Mazcko , “Improving Human Cancer Therapy Through the Evaluation of Pet Dogs,” Nature Reviews Cancer 20, no. 12 (2020): 727–742, 10.1038/s41568-020-0297-3. 32934365

[50] Y. Pekcevik , M. O. Kahya , and A. Kaya , “Characterization of Soft Tissue Tumors by Diffusion‐Weighted Imaging,” Iranian Journal of Radiology 12, no. 3 (2015): 15478, 10.5812/iranjradiol.15478v2. PMC4632135 26557269

[51] C. Messina , R. Bignone , A. Bruno , et al., “Diffusion‐Weighted Imaging in Oncology: An Update,” Cancers 12, no. 6 (2020): 1493, 10.3390/cancers12061493. PMC7352852 32521645

[52] T. Robba , V. Chianca , D. Albano , et al., “Diffusion‐Weighted Imaging for the Cellularity Assessment and Matrix Characterization of Soft Tissue Tumour,” La radiologia medica 122, no. 11 (2017): 871–879, 10.1007/s11547-017-0787-x. 28689283

[53] T. K. Subhawong , M. A. Jacobs , and L. M. Fayad , “Insights Into Quantitative Diffusion‐Weighted MRI for Musculoskeletal Tumor Imaging,” American Journal of Roentgenology 203, no. 3 (2014): 560–572, 10.2214/AJR.13.12165. 25148158

[54] Y. J. Choi , I. S. Lee , Y. S. Song , J. I. Kim , K. U. Choi , and J. W. Song , “Diagnostic Performance of Diffusion‐Weighted (DWI) and Dynamic Contrast‐Enhanced (DCE) MRI for the Differentiation of Benign From Malignant Soft‐Tissue Tumors,” Journal of Magnetic Resonance Imaging 50, no. 3 (2019): 798–809, 10.1002/jmri.26607. 30663160

[55] K. Engin , D. B. Leeper , J. R. Cater , A. J. Thistlethwaite , L. Tupchong , and J. D. McFarlane , “Extracellular pH Distribution in Human Tumours,” International Journal of Hyperthermia 11, no. 2 (1995): 211–216, 10.3109/02656739509022457. 7790735

[56] D. M. Prescott , H. C. Charles , J. M. Poulson , et al., “The Relationship Between Intracellular and Extracellular pH in Spontaneous Canine Tumors,” Clinical Cancer Research 6 (2000): 2501–2505. 10873105

[57] Z. Bhujwalla , C. McCoy , J. Glickson , R. GiIlies , and M. Stubbs , “Estimations of Intra‐ and Extracellular Volume and pH by ^31^P Magnetic Resonance Spectroscopy: Effect of Therapy on RIF‐1 Tumours,” British Journal of Cancer 78 (1998): 606–611, 10.1038/bjc.1998.548. PMC2063062 9744499

[58] M. Lora‐Michiels , D. Yu , L. Sanders , et al., “Extracellular pH and P‐31 Magnetic Resonance Spectroscopic Variables are Related to Outcome in Canine Soft Tissue Sarcomas Treated With Thermoradiotherapy,” Clinical Cancer Research 12, no. 19 (2006): 5733–5740, 10.1158/1078-0432.CCR-05-2669. 17020978

[59] P. E. Z. Larson , H. Y. Chen , J. W. Gordon , et al., “Investigation of Analysis Methods for Hyperpolarized ^13^C‐Pyruvate Metabolic MRI in Prostate Cancer Patients,” NMR in Biomedicine 31, no. 11 (2018): 3997, 10.1002/nbm.3997. PMC6392436 30230646

[60] G. J. Topping , C. Hundshammer , L. Nagel , et al., “Acquisition Strategies for Spatially Resolved Magnetic Resonance Detection of Hyperpolarized Nuclei,” Magnetic Resonance Materials in Physics, Biology and Medicine 33 (2020): 221–256, 10.1007/s10334-019-00807-6. PMC7109201 31811491

[61] J. G. Skinner , G. J. Topping , L. Nagel , et al., “Spectrally Selective bSSFP Using Off‐Resonant RF Excitations Permits High Spatiotemporal Resolution 3D Metabolic Imaging of Hyperpolarized [1‐^13^C]Pyruvate‐to‐[1‐^13^C]lactate Conversion,” Magnetic Resonance in Medicine 90 (2023): 894–909, 10.1002/mrm.29676. 37093981

[62] M. L. Taddei , L. Pietrovito , A. Leo , and P. Chiarugi , “Lactate in Sarcoma Microenvironment: Much More Than just a Waste Product,” Cells 9, no. 2 (2020): 510, 10.3390/cells9020510. PMC7072766 32102348

[63] J. H. Shaw , D. M. Humberstone , and R. R. Wolfe , “Energy and Protein Metabolism in Sarcoma Patients,” Annals of Surgery 207, no. 3 (1988): 283–289, 10.1097/00000658-198803000-00010. PMC1493415 3422801

[64] K. Allemann , M. T. Wyss , M. Wergin , et al., “Measurements of Hypoxia ([^18^F]‐FMISO, [^18^F]‐EF5) With Positron Emission Tomography (PET) and Perfusion Using PET ([^15^O]‐H_2_O) and Power Doppler Ultrasonography in feline fibrosarcomas*,” Veterinary and Comparative Oncology 3, no. 4 (2005): 211–221, 10.1111/j.1476-5810.2005.00081.x. 19754776

[65] J. G. Rajendran , D. C. Wilson , E. U. Conrad , et al., “[^18^F]FMISO and [^18^F]FDG PET Imaging in Soft Tissue Sarcomas: Correlation of Hypoxia, Metabolism and VEGF Expression,” European Journal of Nuclear Medicine and Molecular Imaging 30, no. 5 (2003): 695–704, 10.1007/s00259-002-1096-7. 12632200

[66] K. Shi , C. Bayer , S. T. Astner , et al., “Quantitative Analysis of [^18^F]FMISO PET for Tumor Hypoxia: Correlation of Modeling Results With Immunohistochemistry,” Molecular Imaging and Biology 19, no. 1 (2017): 120–129, 10.1007/s11307-016-0975-4. 27379986

[67] I. Tachibana , Y. Nishimura , K. Hanaoka , et al., “Tumor Hypoxia Detected by ^18^F‐fluoromisonidazole Positron Emission Tomography (FMISO PET) as a Prognostic Indicator of Radiotherapy (RT),” Anticancer Research 38, no. 3 (2018): 1775–1781, 10.21873/anticanres.12415. 29491116

[68] R. Sebro , A. M. Fuller , T. S. Karin Eisenger‐Matheson , et al., “Prospective Exploratory Pilot Study of 18 F‐FMISO PET/CT for Assessing Tumor Hypoxia in Soft Tissue Sarcomas,” Cancer Imaging 26 (2026): 20, 10.1186/s40644-026-01069-x. PMC13536671 42321847

[69] B. Feuerecker , M. Michalik , C. Hundshammer , et al., “Assessment of ^213^Bi‐anti‐EGFR MAb Treatment Efficacy in Malignant Cancer Cells With [1‐^13^C]pyruvate and [^18^F]FDG,” Scientific Reports 9, no. 1 (2019): 8294, 10.1038/s41598-019-44484-w. PMC6549183 31165773

[70] R. L. Hesketh , J. Wang , A. J. Wright , et al., “Magnetic Resonance Imaging Is More Sensitive Than PET for Detecting Treatment‐Induced Cell Death–Dependent Changes in Glycolysis,” Cancer Research 79, no. 14 (2019): 3557–3569, 10.1158/0008-5472.CAN-19-0182. PMC6640042 31088837

[71] S. Yan , C. Zheng , B. Cui , et al., “Multiparametric Imaging Hippocampal Neurodegeneration and Functional Connectivity With Simultaneous PET/MRI in Alzheimer's Disease,” European Journal of Nuclear Medicine and Molecular Imaging 47, no. 10 (2020): 2440–2452, 10.1007/s00259-020-04752-8. PMC7396401 32157432

[72] H. Okazawa , M. Ikawa , M. Jung , et al., “Multimodal Analysis Using [^11^C]PiB‐PET/MRI for Functional Evaluation of Patients With Alzheimer's Disease,” EJNMMI Research 10, no. 1 (2020): 30, 10.1186/s13550-020-00619-z. PMC7105527 32232573

[73] D. Biel , Y. Luan , M. Brendel , et al., “Combining tau‐PET and fMRI Meta‐Analyses for Patient‐Centered Prediction of Cognitive Decline in Alzheimer's Disease,” Alzheimer's Research & Therapy 14, no. 1 (2022): 166, 10.1186/s13195-022-01105-5. PMC9639286 36345046

[74] W. Li , J. Lu , K. Xiao , et al., “Integrated Multimodality Microscope for Accurate and Efficient Target‐Guided Cryo‐Lamellae Preparation,” Nature Methods 20, no. 2 (2023): 268–275, 10.1038/s41592-022-01749-z. PMC9911353 36646896

[75] A. Drzezga , M. Souvatzoglou , M. Eiber , et al., “First Clinical Experience With Integrated Whole‐Body PET/MR: Comparison to PET/CT in Patients With Oncologic Diagnoses,” Journal of Nuclear Medicine 53, no. 6 (2012): 845–855, 10.2967/jnumed.111.098608. 22534830

[76] W. T. Dixon , “Simple Proton Spectroscopic Imaging,” Radiology 153, no. 1 (1984): 189–194, 10.1148/radiology.153.1.6089263. 6089263

[77] M. A. Griswold , P. M. Jakob , R. M. Heidemann , et al., “Generalized Autocalibrating Partially Parallel Acquisitions (GRAPPA),” Magnetic Resonance in Medicine 47, no. 6 (2002): 1202–1210, 10.1002/mrm.10171. 12111967

[78] A. Haase , J. Frahm , D. Matthaei , W. Hanicke , and K. D. Merboldt , “FLASH Imaging. Rapid NMR Imaging Using Low Flip‐Angle Pulses,” Journal of Magnetic Resonance (1969) 67, no. 2 (1986): 258–266, 10.1016/0022-2364(86)90433-6. 22152368

[79] D. J. Meuten , Tumors in Domestic Animals (Wiley, 2016), 10.1002/9781119181200.

[80] K. Dittmer , P. Roccabianca , and C. Bell , Surgical Pathology of Tumors of Domestic Animals Volume 4: Tumors of Bone, Cartilage and Other Hard Tissues (Davis‐Thompson DVM Foundation, 2021).

[81] I. N. Fleming , R. Manavaki , P. J. Blower , et al., “Imaging Tumour Hypoxia With Positron Emission Tomography,” British Journal of Cancer 112, no. 2 (2015): 238–250, 10.1038/bjc.2014.610. PMC4453462 25514380

[82] C. S. Patlak , R. G. Blasberg , and J. D. Fenstermacher , “Graphical Evaluation of Blood‐to‐Brain Transfer Constants From Multiple‐Time Uptake Data,” Journal of Cerebral Blood Flow & Metabolism 3, no. 1 (1983): 1–7, 10.1038/jcbfm.1983.1. 6822610

[83] P. Bankhead , M. B. Loughrey , J. A. Fernandez , et al., “QuPath: Open Source Software for Digital Pathology Image Analysis,” Scientific Reports 7 (2017): 16878, 10.1038/s41598-017-17204-5. PMC5715110 29203879

[84] I. T. Jolliffe and J. Cadima , “Principal Component Analysis: A Review and Recent Developments,” Philosophical Transactions of the Royal Society A: Mathematical, Physical and Engineering Sciences 374, no. 2065 (2016): 20150202, 10.1098/rsta.2015.0202. PMC4792409 26953178

[85] A. Demircioğlu , “The Effect of Feature Normalization Methods in Radiomics,” Insights into Imaging 15, no. 1 (2024): 2, 10.1186/s13244-023-01575-7. PMC10772134 38185786

[86] G. P. McCabe , “Principal Variables,” Technometrics 26, no. 2 (1984): 137–144, 10.1080/00401706.1984.10487939.

[87] W. J. Krzanowski , “Selection of Variables to Preserve Multivariate Data Structure, Using Principal Components,” Applied Statistics 36, no. 1 (1987): 22, 10.2307/2347842.

[88] Y. Mori , Y. Tanaka , and T. Tarumi , “Principal Component Analysis Based on a Subset of Variables for Qualitative Data,” in Data Science, Classification, and Related Methods (Springer, 1998), 547–554, 10.1007/978-4-431-65950-1_60.

