# Supplementary material for: Multimodal Imaging of Hypoxia, Metabolism and pH in Feline Sarcoma Using Hyperpolarized 13C MRI and PET
Source: Adv Sci (Weinh). 2026 Sep 27:e77830. Online ahead of print. doi: 10.1002/advs.77830 (PMC13616244; doi:10.1002/advs.77830)
Supplement: Supplementary file 1 [file ADVS-9999-e77830-s001.docx]

**Supporting information**

**Multimodal imaging of hypoxia, metabolism and pH in feline sarcoma using hyperpolarized ^13^C MRI and PET**

*Martin Grashei, Tanja Groll, Stephanie Kahl, Silke Baer, Elisabeth Bliemsrieder, Frits H. A. van Heijster, Andre Wendlinger, Pascal Wodtke, Jorge Cabello, Susan Notohamiprodjo, Christian Lohrmann, Markus Schwaiger, Wolfgang A. Weber, Katja Steiger, Andrea Meyer-Lindenberg, Johannes Hirschberger, Christine Baumgartner^#^, Franz Schilling^#*^*

^#^These authors contributed equally

Corresponding author Email: schilling@tum.de

**Table S1. Overview of patients being included in this study and respective tumor characteristics. M: male; F: female; n: neutered, r: right, l: left.**

**Fig. S1. Overview of anaesthesia monitoring. (A)** Following initiation of anaesthesia (t = 0 min), six anaesthesia parameters (heart rate, endtidal CO_2_, peak inspiratory pressure, peripheral oxygen saturation (SpO_2_), respiratory minute volume and applied isoflurane fraction in anaesthesia gas) were monitored every 10 minutes for each animal. Dashed lines indicate the time points of injection (FMISO: [^18^F]FMISO injection; ZA: [1,5-^13^C_2_,3,6,6,6-D_4_]zymonic acid injection; PA: [1-^13^C]pyruvate injection). No adverse changes in monitoring parameters can be observed. **(B)** – **(G)** Summary plots of all animals for each parameter in (A). Grey shaded areas indicate session intervals during which all respective agent administrations occurred. Reference intervals for each parameter are indicated by red horizontal lines or extend beyond the plotted ranges. Reference intervals: Heart rate: 100 – 180 1/min; endtidal CO_2_: 20 – 60 mmHg; peak inspiratory pressure: 0 – 20 cm H_2_O; peripheral oxygen saturation: 95 – 100 %; respiratory minute volume: 0.5 – 1.7 l/min; isoflurane concentration: 0.5 – 3 %.

**Fig. S2. Overview of blood count data. (A)** 14 standard blood count parameters and 13 serum parameters were evaluated for blood samples drawn from feline sarcoma patients during health checks prior to surgery, immediately before and after imaging, as well as prior to and post surgery. Day 0 denotes the day of the multimodal imaging session. Not all timepoints are recorded for all patients and intervals prior and post imaging vary between patients. **(B)** Significant changes in several blood count parameters post imaging compared to baseline values before imaging are detectable, with only white blood cells exceeding the reference range. **(C)** Comparison of a subset of samples being drawn immediately prior and post imaging show only two blood count markers and serum marker to be altered. Here, white blood cell values and three serum values exceed reference ranges post imaging, due to injection of a urea-containing buffered solution. Together, no findings beyond a mild stress reaction are observed, indicating the tolerability of all injections within the protocol.

**Fig. S3. Comparison of muscle and blood pH compartments measured by MRI as tissue pH reference.** Tumour-adjacent muscle and central blood-vessel ROIs both are suitable as a consistent tissue pH reference in hyperpolarized ¹³C MRSI, while absolute pH values vary with subject and anaesthesia.
**Fig. S4. Hypoxia validation by quantification of pimonidazole immunohistochemical stainings. (A)** Exemplary pimonidazole immunohistochemical section of a FS G3. **(B)** Exemplary area (magnification 40x) from (A), showing strong heterogeneous presence of hypoxia indicated by pimonidazole positivity (brown diaminobenzidine precipitate. **(C)** Section from (A) overlaid with cell detections using QuPath. Red and blue areas indicate hypoxia-positive and -negative areas respectively, using a binary classification threshold. **(D)** Exemplary area from (B) overlaid with single cell detections, each consisting of two ROIs, delineating the cell nucleus and the expansion of the cell. The position of the magnification in (A) and (C) is indicated with yellow arrows. **(E)** Control section of a FS without pimonidazole injection showing virtually no unspecific staining and classification (0.25% absolute false positive detections). **(F)** Exemplary area (magnification 40x) from (E). **(G)** Parameters for positive cell detection with binary classification. Neither PET-derived SUV **(H)** nor K_i_ **(I)** show a correlation with pimonidazole-positive cell fraction. Scale bars in (B), (D), (F): 20 µm.

**Fig. S5. Questionnaire for follow-up survey on feline sarcoma patient outcome.**

**Fig. S6. Correlation of imaging parameters and patient overall survival.** Neither static **(A)** nor kinetic **(B)** PET parameters show a relevant correlation with patient overall survival. Increasing ADC values **(C)** and higher metabolic activity **(D)** show a non-significant correlation with shorter survival. pH **(E)** and acidification **(F)** reveal the strongest correlation with a reduction in overall survival, however, neither correlation is statistically significant. Notably, the two patients bearing the tumors with strongest acidifications exhibit the shortest overall survival of all surveyed patients.

**Fig. S7. Survival and correlation analysis including subject meta parameters. (A)** Tumors volumes, **(B)** subject age, **(C)** weight, **(D)** sex, **(E)** breed and **(F)** tumor location were assessed as predictive markers for patient outcome. For continuous variables **(A-C)**, cutoff values were determined analogous to analysis in Figure 9. For nominal variables (**D-F**), groups were split into male and female for sex, European domestic and non-european domestic for breed and tumors being located on the abdominal wall or at another location. None of the assessed parameters show a significant prognostic potential. **(G)** To assess confounding factors for survival analysis, age, body weight (BW) and tumor volume were pairwise correlated with image-derived parameters. Tumor size (*r* = 0.51, *p* = 0.03) and SUV (*r* = 0.56, *p* = 0.03) correlate significantly (bold-framed) negative with subject age. Tumor volume correlates positive with ADC (r = 0.77, *p* = 0.0005) and metabolic activity (*r* = 0.77, *p* = 0.0008). **(H)** When dichotomizing the subject cohort according to sex (male vs female), tumor subtype (fibrosarcoma vs. non-fibrosarcoma), breed (European domestic vs. non-european domestic) and tumor location (abdominal wall vs. other locations), male subjects show a higher body weight (*p* = 0.02) and tumors located on the abdominal wall appear significantly less acidified (*p* = 0.02) compared to other tumor locations. *p* values were not adjusted for multiple comparisons.

**Fig. S8. Animated rotation of Fig. 6G.**

**Fig. S9. Animated rotation of Fig. 6H.**

**Fig. S10. Animated rotation of Fig. 7C.**

**Fig. S11. Animated rotation of Fig. 7D.**

**Fig. S12. Animated rotation of Fig. 8D.**
